# Protocols for yTREX/Tn5‐based gene cluster expression in *Pseudomonas putida*

**DOI:** 10.1111/1751-7915.13402

**Published:** 2019-06-04

**Authors:** Robin Weihmann, Andreas Domröse, Thomas Drepper, Karl‐Erich Jaeger, Anita Loeschcke

**Affiliations:** ^1^ Institute of Molecular Enzyme Technology Heinrich‐Heine‐University Düsseldorf Forschungszentrum Jülich Jülich Germany; ^2^ Institute of Medical Microbiology and Hospital Hygiene Heinrich‐Heine‐University Düsseldorf Germany

## Abstract

Bacterial gene clusters, which represent a genetic treasure trove for secondary metabolite pathways, often need to be activated in a heterologous host to access the valuable biosynthetic products. We provide here a detailed protocol for the application of the yTREX ‘gene cluster transplantation tool’: *Via* yeast recombinational cloning, a gene cluster of interest can be cloned in the yTREX vector, which enables the robust conjugational transfer of the gene cluster to bacteria like *Pseudomonas putida*, and their subsequent transposon Tn5‐based insertion into the host chromosome. Depending on the gene cluster architecture and chromosomal insertion site, the respective pathway genes can be transcribed effectively from a chromosomal promoter, thereby enabling the biosynthesis of a natural product. We describe workflows for the design of a gene cluster expression cassette, cloning of the cassette in the yTREX vector by yeast recombineering, and subsequent transfer and expression in *P. putida*. As an example for yTREX‐based transplantation of a natural product biosynthesis, we provide details on the cloning and activation of the phenazine‐1‐carboxylic acid biosynthetic genes from *Pseudomonas aeruginosa* in *P. putida* KT2440 as well as the use of *β‐*galactosidase‐encoding *lacZ* as a reporter of production levels.

## Graphical abstract

A detailed protocol for the application of the yTREX ‘gene cluster transplantation tool’ is provided: We describe workflows for the design of a gene cluster expression cassette, cloning of the cassette in the yTREX vector by yeast recombineering, and subsequent transfer and expression in *Pseudomonas putida*. Cloning and activation of the phenazine‐1‐carboxylic acid biosynthetic genes from *Pseudomonas aeruginosa* in *P. putida* KT2440 and the use of *β*‐galactosidase‐encoding *lacZ* as a reporter of production levels is demonstrated as an application.

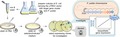

## Introduction

Bacterial secondary metabolites represent an invaluable source of high‐value compounds with diverse applications, for example in the pharma or agricultural sector (Vaishnav and Demain, 2010; Katz and Baltz, 2016). However, certain challenges hamper straightforward access to these compounds: the natural producers often synthesize the compound of interest in low amounts or in very complex mixtures with other compounds (Rutledge and Challis, 2015). Moreover, an up‐scaled bioreactor cultivation of environmental bacteria may be associated with safety issues or simply not possible because the microbe would not grow under laboratory conditions. Therefore, heterologous expression of clustered biosynthetic genes in an amenable expression host often represents a reasonable alternative, but may bring other challenges: cloning of large DNA fragments is often tedious, and the stable maintenance of heterologous biosynthetic genes and their effective expression in the host are often difficult to implement. Moreover, the production of bioactive secondary metabolites such as antibiotic compounds can impair strain stability and compromise product yields.

Therefore, appropriate microbial chassis and molecular genetic tools are essential for the successful heterologous metabolite production (Kim *et al*., 2015; Zhang *et al*., 2016). *Pseudomonas* species exhibit a remarkable tolerance to xenobiotics rendering them especially suitable as microbial cell factories for small molecule natural compounds (Nikel *et al*., 2014, 2016; Loeschcke and Thies, 2015; Nikel and de Lorenzo, 2018). We have previously introduced the pathway transfer and expression (TREX) system and its application for gene cluster expression in different bacterial hosts including *Pseudomonas putida* KT2440 (Loeschcke *et al*., 2013).

The TREX tool basically consists of a vector harbouring all genetic elements necessary for the straightforward transfer and expression of gene clusters in host bacteria. Possible applications of the tool include the ‘transplantation’ of known biosynthetic pathways for the generation of metabolite production strains or the assessment of a host's suitability in this regard. Furthermore, the TREX tool can be employed for the transfer and expression of yet uncharacterized biosynthetic genes for the elucidation of their function and biosynthetic product. Key steps of the TREX procedure are the cloning of a gene cluster of interest together with the TREX cassettes in one vector, the transfer and random transposon Tn5‐based chromosomal integration in the host strain, and the expression *via* the host or T7 RNA polymerase. The system was recently updated and named yTREX, now enabling yeast recombination‐based assembly cloning of a target gene cluster into a designated site of the yTREX vector (Domröse *et al*., 2017). Applicability was demonstrated in a proof of concept study using the prodigiosin, violacein and phenazine pathways for the assembly and expression of five customized gene clusters in *P. putida* KT2440 (Domröse *et al*., 2017). During our applications in that study and in further experiments, we have observed that (i) the random chromosomal integration can lead to the identification of especially suitable sites for gene cluster expression (Domröse *et al*., 2015; A. Domröse and A. Loeschcke unpublished) and (ii) the use of a simple and robust transcription reporter can indicate the transcription of biosynthetic genes (Domröse *et al*., 2017). The yTREX tool can be applied in laboratories with standard equipment for microbiology/molecular genetic work. The yTREX vector is distributed by our laboratory upon request, and the corresponding DNA sequence is included in the supporting information of this manuscript and deposited at the NCBI database (GenBank MK416190).

In the following protocols, we describe the yTREX workflow starting with the design of a gene cluster expression project, subsequent cloning of the biosynthetic gene cluster facilitated by yeast recombineering, expression in *P. putida* and identification of suitable metabolite producing strains. The procedure is exemplified by the activation of phenazine biosynthetic genes in *P. putida* KT2440 and the use of *lacZ* as reporter for gene expression and production.

## Step‐by‐step yTREX application

### Design of the expression construct

**Figure 1:**
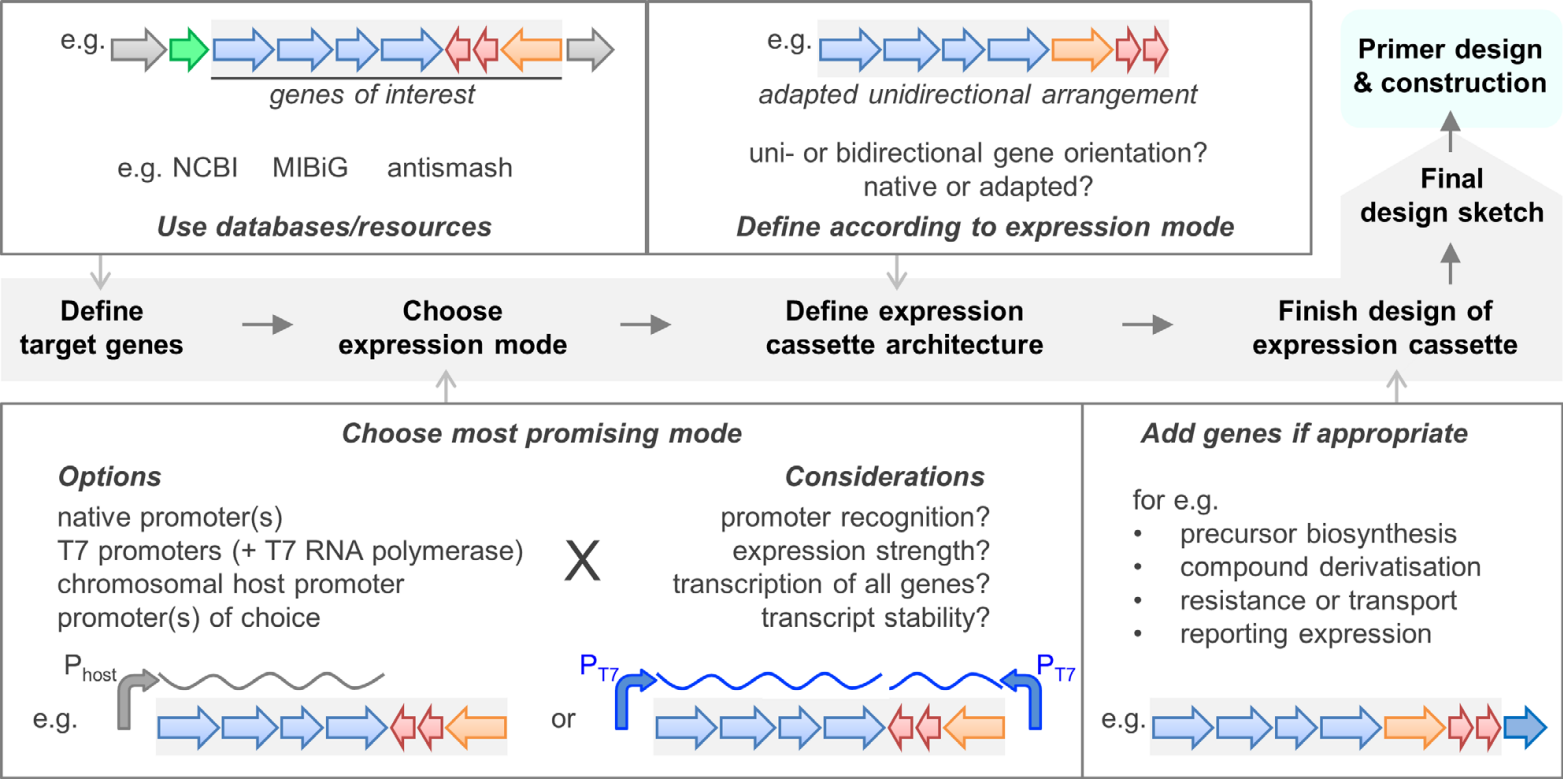
Workflow for designing a yTREX‐based gene cluster expression cassette. The target genes of interest must be defined, for example using diverse databases and resources. For the defined target genes, an expression mode is chosen, considering possible advantages or pitfalls associated with each option (see also Table 1). This choice further guides the definition of the gene cluster expression cassette architecture (e.g. genes are required to be organized in unidirectional arrangement without transcription termination sites to enable expression from a single promoter). The design sketch can finally be extended by additional elements like a reporter gene, before primer design and construction.

The yTREX application is based on the assembly of a target gene cluster or a set of genes of interest in the yTREX vector and its transfer into a bacterial host for expression. Depending on the final construct to be generated and the planned expression mode for the target genes, multiple aspects should be considered for the design of a suitable ‘gene cluster expression cassette’ (i.e. all elements of a naturally occurring or a synthetically assembled gene cluster which shall be cloned in the yTREX vector and expressed in a host bacterium). We have preferentially chosen to implement gene cluster expression *via* a chromosomal host promoter after random chromosomal integration. Nevertheless, we also provide here an overview on multiple options and a more general workflow for designing an appropriate expression cassette (Fig. 1).

Step 1: As a first step, define the genes or gene cluster to be cloned and expressed. This step is certainly highly dependent on the specifics of the given project and difficult to generalize, but of great importance and thus underlined. Basically, to ensure that the entire gene cluster is targeted including all relevant biosynthetic genes, double‐checking of annotations (if available) making use of resources like MIBiG https://mibig.secondarymetabolites.org/ (Medema *et al*., 2015), antiSMASH at http://antismash.secondarymetabolites.org (Medema *et al*., 2011; Blin *et al*., 2017) or NCBI at https://www.ncbi.nlm.nih.gov/ (NCBI Resource Coordinators, 2017) and respective homology search tools is recommended. During this step, also codon usage compatibility of the gene cluster and the expression host (here *P. putida* KT2440) should be assessed, for example by using the graphical codon usage analyser at http://gcua.schoedl.de/ (Fuhrmann *et al*., 2004) and the Kazusa Codon Usage Database at https://www.kazusa.or.jp/codon/ (Nakamura *et al*., 2000).
Step 2: Choose an expression mode and define the expression cassette architecture accordingly. Different alternative promoter systems and associated modes of expression may be considered, each influencing the design of the gene cluster expression cassette, and each associated with specific potential pitfalls (summarized in Table 1).
  **Table 1:** Summary of modes to be considered for the expression of gene clusters in a bacterial host. Corresponding possible or necessary gene cluster architectures and associated potential pitfalls are listed for each case

Planned expression mode	Possible cluster architecture	Potential pitfalls to be considered	Reference with TREX example
Native gene cluster promoter(s) and host RNA polymerase	Irrespective of gene cluster architecture >Use of native cluster possible	Weak or incomplete expression because of incomplete promoter recognition; limited in the modulation of expression levels	Loeschcke *et al*. (2013)
One chromosomal host promoter and host RNA polymerase	Must be one transcriptional unit >Native or needs to be adapted	Transcription stop at (unknown) terminators within native gene clusters; gene cluster adaptation may compromise native functionality (e.g. transcript stability)	Domröse *et al*. (2015, 2017)
Two T7 promoters at ends of the gene cluster (in (y)TREX cassettes) and T7 RNA polymerase	Irrespective of gene cluster architecture >Use of native cluster possible	Additional burden of T7 RNA polymerase expression; lowered expression efficiency due to convergent bidirectional expression	Loeschcke *et al*. (2013)
One bacterial or phage‐derived promoter of choice and host or T7 RNA polymerase	Can be chosen >Adaptation (at least promoter addition) required	For host polymerase: Transcription stop at (unknown) terminators within gene clusters; gene cluster adaptation may compromise native functionality (e.g. transcript stability)	S. Kubicki, S. Thies and A. Loeschcke unpublished

- The simplest option is to rely on the recognition of the native promoters of the gene cluster by the host RNA polymerase, ideally leading to the concerted transcription of all pathway genes. This option is viable completely irrespective of the native gene cluster architecture and requires no adaptation, only the inclusion of the respective original promoter regions (Loeschcke *et al*., 2013). However, depending on the origin of the genes, the native regulatory network and the chosen host, this approach can fail. Moreover, it does not allow testing of different expression levels to optimize the yield of the biosynthetic product.
- For gene clusters which are naturally organized as a single unidirectional transcriptional unit, we recommend to aim at random chromosomal insertion downstream of a suitable host promoter which can lead to the expression of all pathway genes. This approach facilitates the reliable generation of robust expression strains (Domröse *et al*., 2015, 2017) and testing of various host promoters and expression levels, as outlined in the following step‐by‐step guide and demonstrated in the application example given below. It moreover enables the identification of chromosomal sites like the rRNA‐encoding *rrn* operons that are particularly suitable for the expression of clustered genes (Domröse *et al*., 2015; A. Domröse and A. Loeschcke, unpublished).
- The expression of all genes within a cluster, which exhibits a complex organization with multiple transcriptional units with bidirectional gene orientation, can be achieved by convergent T7 RNA polymerase‐based expression (Loeschcke *et al*., 2013). However, the T7 RNA polymerase gene is not included in the yTREX transposon and thus has to be additionally expressed in another way, that is by using one of the available strains carrying the respective polymerase gene in the chromosome or by plasmid‐based expression, for example using pML5‐T7 (Arvani *et al*., 2012; Loeschcke *et al*., 2013). In any case, expression of T7 RNA polymerase brings about an extra burden for the host. Note that use of the yTREX vector always entails this option because it contains the respective promoter sequences in the L‐ and R‐yTREX cassettes which can be readily used for expression (find further information on the composition of the yTREX cassettes in section Generation of gene cluster DNA fragments & yeast assembly cloning in the yTREX vector and the Table S1). Therefore, this may represent a backup solution if another concept [like option (i)] fails to implement effective gene expression and pathway biosynthesis. Alternatively, a complex gene cluster that is composed of multiple transcriptional units can be re‐arranged into unidirectional format without (putative) transcription termination sites to enable option (ii). While this is relatively easy to realize during assembly cloning, it is very difficult to predict if manipulations of the original sequence may compromise transcription, the native transcript stability or translation efficiency.
- During cloning, also one or more promoters of choice may be included to direct gene expression. If the appropriate knowledge on promoter functionalities in the host, which is to be used, is available, this option enables to specifically install different expression levels. Note that the addition of the same promoter sequence at several sites should be avoided if assembly cloning is applied to avoid unwanted recombination events. The addition of a single promoter can be combined with a re‐arrangement into a single transcriptional unit if the gene cluster of interest does not naturally exhibit this architecture.

Step 3: Consider including additional elements. Depending on the project, additional precursor biosynthetic genes or genes encoding derivatizing enzymes or transporters that secrete the biosynthetic product might be of interest (not further addressed in this protocol article). For the facile identification of clones expressing the gene cluster in later steps, a promoter‐less reporter gene encoding a protein with known functionality in the host strain may be added. In our hands, among others, *β*‐galactosidase‐encoding *lacZ* from *Escherichia coli* represents a very robust reporter gene for use in *P. putida* KT2440 (see section yTREX gene cluster transfer, transposon integration & implementation of gene expression). Fluorescent proteins may be considered as alternative reporters.

### Generation of gene cluster DNA fragments and yeast assembly cloning in the yTREX vector

**Figure 2:**
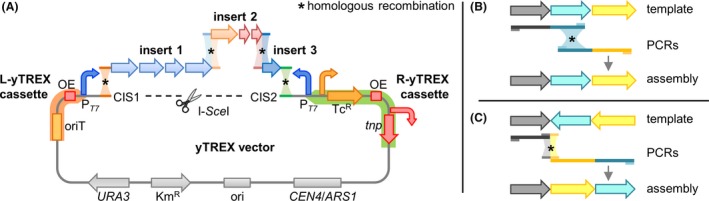
Schematic of the gene cluster assembly in the yTREX vector. A. The yTREX vector backbone comprises replication elements and selection markers for *E. coli* (ori, pMB1 origin of replication; Km^R^, kanamycin resistance gene) and yeast (*CEN4*/*ARS1*, *S. cerevisiae* centromere region and autonomously replicating sequence; *URA3*, orotidine 5′‐phosphate decarboxylase gene) and the yTREX cassettes. L‐yTREX (orange): oriT, origin of transfer; OE, outside end of transposon Tn5; P_*T*__*7*_, T7 bacteriophage promoter, R‐yTREX (green): *tnp*, Tn5 transposase gene; OE; Tc^R^, tetracycline resistance gene; P_*T*__*7*_. The vector is linearized by hydrolysis with restriction endonuclease I‐*Sce*I, thereby exposing the partial I‐*Sce*I recognition site and the sequences of the CIS (cluster integration site) at the termini. At the respective CIS1 and CIS2 sequences, insert fragments with appropriate homology arms to the CIS sequences and to one another can be integrated *via* yeast recombineering. Depiction is not drawn to scale. The complete vector sequence is available at the NCBI database (GenBank MK416190) and in the Table S1 in GenBank format. Right panel: Creation of homologous regions for recombination can generally be achieved by PCR and appropriate positioning of fully binding primers. Accordingly, designed primers can be used to re‐assemble large gene clusters in their native organization from freely defined PCR fragments (B). Alternatively, the use of primers with 5′‐elongations adding sequences to match new adjacent fragments enables re‐arrangements of genes or the addition of new parts (C). In this case, primer positions are defined by the ends of the fragments that are to be connected. Find further information under section Generation of gene cluster DNA fragments & yeast assembly cloning in the yTREX vector, step 3b.

Various molecular genetic methods may be applied to assemble the expression construct. We describe here the methodology for yeast recombinational cloning. This enables the integration of multiple DNA fragments into the yTREX vector *via* homologous sequences (Fig. 2). To this end, appropriate DNA fragments need to be generated which is typically achieved *via* PCR and described in the following section. The methodology of yeast recombinational gene cluster cloning has been described before (e.g. Shao and Zhao, 2014); we therefore focus on details specific for our workflow using the yTREX vector and primer design for the assembly of synthetic gene clusters.

Step 1: To linearize the yTREX vector by hydrolyzation using I‐*Sce*I restriction endonuclease, prepare a 30 μl reaction mixture with 1 μl I‐*Sce*I (5 U), the manufacturer's buffer (New England Biolabs GmbH, Ipswich, USA) as well as ˜2 μg vector DNA, and incubate for 16 h at 37°C (according to the manufacturer's specifications, a lower amount of enzyme (1–2 U) would also be sufficient for this step). Subsequently, subject the resulting linearized vector to treatment with a phosphatase like FastAP (Thermo Fisher Scientific GmbH, Walkham, UT, USA) by addition of 1 μl of the enzyme to the I‐*Sce*I‐reaction mixture and incubation for further 30 min at 37°C to avoid re‐circularization of the empty vector by ligase enzymes in yeast cells (Suzuki *et al*., 1983; Wilson *et al*., 1997) during assembly cloning in later steps. Finally, inactivate both enzymes by incubation at 75°C for 10 min.
Step 2: Based on the final design sketch of the expression cassette (see section Design of the expression construct), which will be assembled, broadly divide the gene cluster into PCR sections before the exact positioning of primers in the next steps. Here, a balance has to be found between the number of fragments and fragment lengths, which can both impair assembly efficiency (Shao and Zhao, 2014). In our hands, PCR fragments are typically planned to be up to 8 kb length. The generation and use of longer fragments is also possible and has been described before (Gibson *et al*., 2008; Shao *et al*., 2011; Bilyk *et al*., 2016). However, one should note that the probability of sequence errors during PCR rises with increasing product size. In that case, use of ‘high‐fidelity’ DNA polymerases is strongly recommended. Genes whose orientation has to be adapted according to the design concept of the expression cassette (see section Design of the expression construct) need to be amplified separately to other sections (see scheme in Fig. 2C).
Step 3: Design primers for PCR amplification of target genes: Since the design of primers is specific for every gene cluster of interest, only general guidelines are given here describing feasible procedures and potential pitfalls.
Step 3a: Design primers for yeast recombineering of fragments with the yTREX vector (Fig. 2) according to the following general guidelines: The primer binding to the 5′‐UTR (untranslated region including the RBS) upstream of the first gene of the target cluster has to be elongated (at the 5′‐end of the primer) by adding a sequence enabling homologous recombination with the CIS sequence of the L‐yTREX cassette (CIS1). The primer binding to the 3′‐end of the last gene of the target cluster has to be elongated with the sequence enabling homologous recombination with the CIS sequence of the R‐yTREX cassette (CIS2) (use the reverse and complementary sequence of the here given sequence to add to a primer that is written from 5′‐ to 3′‐end).

CIS1, 5′‐CCTCCAAACTAGAAATATTAGCTAATTTAATCTCTCAACC‐3′

CIS2, 5′‐TTGGCGTAATCATGGTCATAGCTGTTTCCTGTGTGAAATT‐3′

We have typically used approximately 30 bp elongations of primers homologous to CIS sequences (underlined parts), and this has worked for yeast recombineering‐based integration of a relatively large fragment (> 20 kb) into the yTREX vector (Domröse *et al*., 2017). Find a cloning scheme of an application example with a detailed description in Fig. S1. In the case of challenging assemblies (large and/or high number of inserts), longer homology arms can be used to facilitate most efficient yeast recombination (see also below, under section Generation of gene cluster DNA fragments & yeast assembly cloning in the yTREX vector, step 3b). Moreover, primer homology arms can generally be adapted in length and position (within CIS sequences) depending on the sequence binding to the gene cluster of interest in order to obtain suitable overall primer pairs (in terms of GC content and melting temperature).

Step 3b: Design primers for the assembly of multiple insert fragments for yeast recombineering of gene clusters into the yTREX vector according to the following general guidelines: All ends of multiple DNA insert fragments have to be designed to enable assembly of all target genes in the conceptualized order. There are generally two different ways to generate homology arms of fragments *via* primer design, that is *via* the primer positions on the template [see option (i)] or an elongation of the primers at the 5′‐end (see option (ii)] (Fig. 2).

- For the re‐assembly of a gene cluster in its native form from different smaller PCR sections, generate a homology region between two adjacent fragments by positioning the reverse primer binding the 3′‐end of the leading fragment downstream to the position of the forward primer binding the 5′‐end of the following fragment (Fig. 2B). Thereby, a region is generated which is present at the end of both fragments, functioning as homology arms during recombination. This scenario allows the use of relatively small primers (use minimally 20 bp if vector DNA is employed as template, and minimally 25 bp for genomic DNA) and free positioning of primers to determine sizes of homologous regions deliberately (we have typically generated approximately 50 bp overlapping sequences) and to obtain primer sequences with optimal features and melting temperatures (Tm) for an efficient PCR (50–60% GC; *T*_m_ approximately 60°C; last nucleotide G or C). Since the efficiency of recombination is to some extent dependent on the length of the homology arms, longer homology arms should be planned for challenging assemblies of multiple and very long fragments (Manivasakam *et al*., 1995; Oldenburg *et al*., 1997; Raymond *et al*., 1999; Shao and Zhao, 2014; Montiel *et al*., 2015). The limitations of assembly cloning in yeast are basically still unknown, but the assembly of an entire bacterial genome from 25 fragments (approximately 17–35 kb) with 80–360 bp homologous overlaps was demonstrated, indicating the method's potential (Gibson *et al*., 2008).
- For the assembly of a synthetic gene cluster (e.g. if a re‐arrangement of the native structure or a modification of a biosynthetic pathway is planned), the inclusion of non‐template‐binding elongations at the 5′‐ends of the corresponding primers is necessary to generate overhangs homologous to the new adjacent sequences (Fig. 2C). Here, the following points should be considered.

Since the aspired assembly of a specific target sequence largely determines primer sequences (both, the binding sites and the homology arm to the new adjacent fragment, are typically at the ends of genes), common primer design criteria for optimal PCR efficiency are often difficult to fulfil. However, we generally recommend template binding sequences of minimally 20 bp for plasmid templates (minimally 25 bp for genomic DNA) that exhibit a Tm significantly lower than that of the full‐length primer, if possible. Add approximately 30 bp elongations to the 5′‐ends of primers, thereby creating a 30 bp homologous overlap, and up to 60 bp if suitable homology arms are added to both fragments. Overall primer Tm is typically above 72°C due to the total length.

Make sure to include RBS sequences for each gene. Note that in natural gene clusters, often 3′‐ends of coding sequences of upstream genes encompass the RBS or even coding sequences of downstream located genes. This native structure calls for especially careful planning of primers for the assembly of synthetic gene clusters, here outlined for manipulations of the respective downstream gene: If the coding sequences of the upstream and the original downstream genes overlap, add the new downstream gene together with its own RBS behind the stop codon of the upstream gene. It might be useful to introduce a stop codon in the reading frame of the former downstream gene before the new one to avoid formation of longer, erroneous peptides. If the coding sequences do not overlap but the RBS of the original downstream gene is located within the coding sequence of the upstream gene, another downstream gene can be added only exchanging the coding sequence from ATG to the stop codon, keeping the former RBS.

For the addition of a reporter gene downstream of the gene cluster, include the coding and RBS sequence of the reporter gene but exclude the promoter from the respective template. The reporter gene has to be connected to the R‐yTREX cassette *via* homologous recombination with the CIS2, and the respective homologous sequence needs to be added to the primer binding at the 3′‐end of the reporter gene (cf. section Generation of gene cluster DNA fragments & yeast assembly cloning in the yTREX vector, step 3a). An exemplary assembly scheme of a recombinant yTREX vector carrying the partial *phz* gene cluster from *Pseudomonas aeruginosa* and the *β*‐galactosidase‐encoding *lacZ* reporter gene (for DNA sequence see Table S2) including details on PCR primer sequences which contain the homologous regions for yeast recombineering is shown in Fig. S1. Any alternative reporter gene can be introduced analogously.

Step 4: Verify correct assembly of the planned yTREX construct *in silico* using appropriate software (e.g. Clone Manager from Sci‐Ed Software), checking integrity of coding sequences, presence of RBSs and completeness of genes (start to stop codon). In this context, check that none of the primers and resulting PCR fragments exhibit long homologies (around 15 or more identical bases) to any non‐target sequences on the designated final plasmid if possible. Such homologies could result in false pairing during homologous recombination.
Step 5: Amplify DNA fragments of the gene cluster of interest *via* PCR with homology arms using appropriate DNA comprising target genes as template, 0.1 μM of each primer and a DNA polymerase with a low error rate like Phusion High‐Fidelity DNA Polymerase (Thermo Fisher Scientific GmbH). Use a PCR programme according to the manufacturer's manual. Note that PCR primers with 5′‐elongations initially bind to the template only partially and thus typically exhibit a significantly lower Tm than the nominal Tm of the entire primer during the first PCR cycles. When the amount of PCR product increases, the nominal Tm applies. This may recommend application of a two‐phased PCR programme with a first phase using an annealing temperature of, for example, 60–65°C for 10 initial cycles, and a second phase using two‐step PCR, that is the alternate implementation of denaturation and elongation temperatures but no specific annealing temperature. Purify PCR products from reaction mixtures to exclude potential unspecific PCR products and template DNA by agarose gel electrophoresis and spin column purification following manufacturers’ protocols (e.g. provided by Analytik Jena AG, Jena, Germany).
Step 6: Assemble yTREX vector and target gene cluster *via* yeast recombineering. The yTREX vector carries the *CEN4*/*ARS1* plasmid replication elements and the *URA3* marker, thus enabling yeast recombination cloning by transformation of DNA fragments with homology arms to one another. *Saccharomyces cerevisiae* transformation protocols have been developed and described previously (Gietzt *et al*., 1995; Gietz and Schiestl, 2007). We therefore included the protocol established in our laboratory in the Appendix S1, mainly aiming at providing additional information on relevant specifics when using the yTREX vector and on slight adaptations we made in the protocols, but also to provide an overview on the complete procedure. Consultation of the original articles including the trouble shooting remarks is highly recommended. Generally, cloning can also be accomplished *via* other methods and researchers may choose to use their preferred procedure like restriction/ligation‐based cloning (which may be problematic in case of large DNA fragments), Gibson assembly (Gibson, 2011) or others (note that choosing another method requires design of appropriate primers).

### yTREX gene cluster transfer, transposon integration and implementation of gene expression

**Figure 3:**
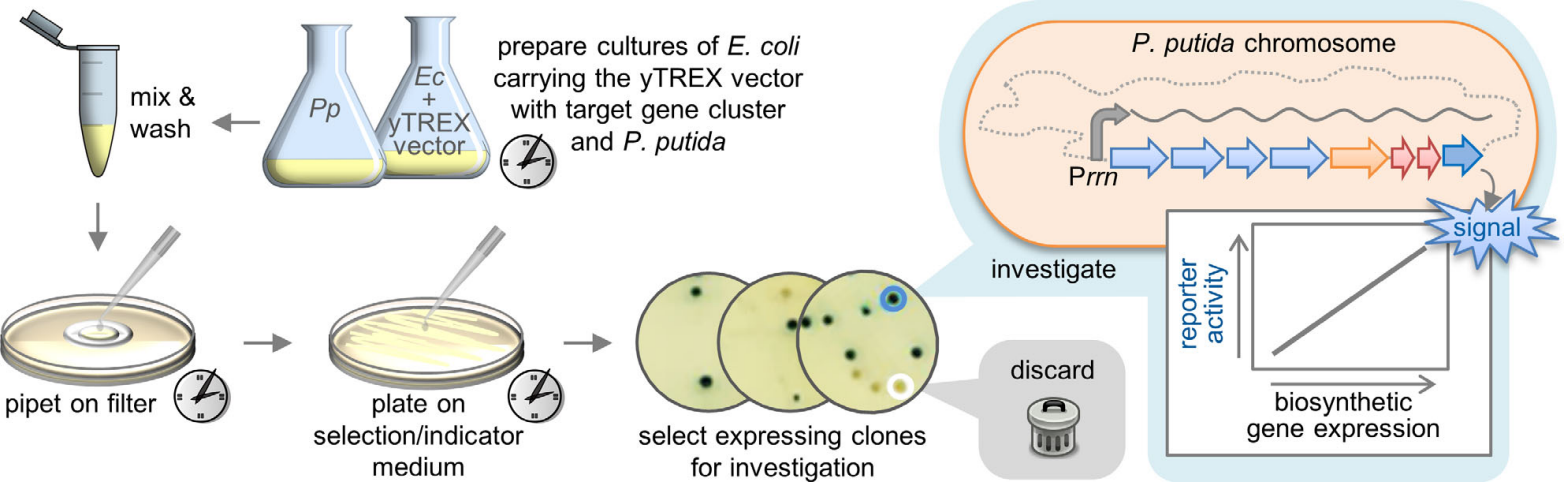
Workflow for yTREX gene cluster transfer, transposon integration & gene expression. Experimental steps of conjugational yTREX vector transfer and growth of transposon‐carrying clones are depicted. Overnight incubations are indicated. The random chromosomal integration of a recombinant yTREX transposon comprising a gene cluster, which exhibits unidirectional gene orientation and no transcription termination sites, can lead to the cluster's transcription by a chromosomal promoter such as P_*rrn*_ of the rRNA‐encoding *rrn* operons (Domröse *et al*., 2015). The addition of a reporter gene like *lacZ* enables selection of such clones by detection of the reporter‐derived signal (Domröse *et al*., 2017). Ideally, the reporter activity is not only related to gene transcription but also related to biosynthetic compound production.

The transfer of a cloned yTREX construct can be achieved *via* conjugation. Researchers may choose to use electroporation of the host instead, relying on established protocols (e.g. using the Eppendorf Eporator^®^ and protocol No. 4308915.529) for *P. putida*. We typically employ conjugational gene transfer using appropriate *E. coli* donor strains as it is a very robust method that reliably transfers also larger DNA constructs. Since the yTREX vector does not replicate in *Pseudomonas*, the vector transfer and growth of recipients exhibiting tetracycline resistance (the respective gene is located within the yTREX Tn5 transposon) exclusively selects recombinant clones carrying the yTREX transposon. The target gene cluster as part of a recombinant yTREX transposon is therefore randomly integrated in the bacterial chromosome after yTREX plasmid transfer to the recipient cells. Thereafter, gene expression can be implemented in different ways (see section Design of the expression construct and Table 1). In the following, we describe the methodology for achieving expression of a unidirectional gene cluster by a host from a chromosomal promoter and selection of such expressing clones by use of the reporter gene *lacZ* (Fig. 3).

Step 1: Transform the previously assembled recombinant yTREX vector into *E. coli* S17‐1 donor strain (Simon *et al*., 1983) *via* commonly established heat shock transformation of chemically competent cells or electroporation. For the case we describe here (aiming at the expression *via* a chromosomal host promoter), the yTREX vector necessarily comprises a unidirectional gene cluster without transcription termination sites which is extended by the reporter gene *lacZ* allowing for the identification of expressing clones.
Step 2: Cultivate the recipient strain like *P. putida* KT2440 and the *E. coli* donor carrying the assembled yTREX vector for conjugation in LB medium, shaking at 30°C (e.g. in Flower Plates obtained from m2p‐labs GmbH, Baesweiler, Germany).
Step 3: Mix donor and recipient cultures (0.25 ml each) in a sterile reaction tube and wash cells by centrifugation (22°C, 1 min, 10 000 *g*), discarding the supernatant and re‐suspending in 1 ml LB medium without antibiotics.
Step 4: To combine cultures for conjugational plasmid transfer, harvest the cells of donor and recipient cultures by centrifugation (22°C, 1 min, 10 000 *g*), discard 800 μl of the supernatant, resuspend the cells carefully with a 1 ml pipette in the residual 200 μl and transfer the mixture on a sterile cellulose acetate membrane filter (0.2 μm pore size, 25 mm diameter by GE Healthcare UK Limited, Buckinghamshire, UK), previously placed on an LB agar plate without antibiotics, and incubate over night at 30°C.
Step 5: To grow transposon‐carrying recipient cells selectively, transfer the filter to a sterile 2 ml reaction tube, add 1 ml LB medium and vortex thoroughly to wash off the cells. Discard the filter and plate 100 μl of the cell suspension on selection medium containing 50 μg ml^−1^ tetracycline and 25 μg ml^−1^ irgasan for the selection of *Pseudomonas* clones carrying the yTREX transposon, and incubate at 30°C for 1–2 days for clone formation. Plate dilutions of cell suspensions if too many clones grow, plate 200 μl or more of the conjugation mixture (in this case, concentrate by centrifugation and re‐suspending cells in a smaller volume of medium) if too few colonies grow. Depending on the biosynthesis which is to be established by gene expression, clones may readily exhibit differential phenotypes (e.g. visible product formation in case of pigment biosynthesis or differential colony sizes). This can indicate in which clones the recombinant transposon was integrated in a chromosomal locus where the transferred gene cluster is expressed from a strong chromosomal host promoter. For additional facile identification of expressing clones by detection of *lacZ*‐encoded *β*‐galactosidase activity, use LB agar plates containing tetracycline, irgasan and Xgal (5‐bromo‐4‐chloro‐3‐indolyl‐*β*‐d‐galactopyranoside), the widely used chromogenic substrate for *β*‐galactosidase. It can be supplemented using a 50 mM stock solution (dissolved, e.g. in DMSO) to a final concentration of 0.3 mM in the medium. Xgal is colourless until cleavage by *β*‐galactosidase, which is followed by dimerization of the indole product, giving the respective clones a blue colour (Horwitz *et al*., 1964). In this case, it is recommended to wash the cells twice with LB medium before plating to avoid any unspecific reporter signal on the plate that may stem from already lysed expressing cells.
Step 6: Use coloured clones for further analysis; if appropriate, *β*‐galactosidase activity may be quantified in selected clones. As the *β*‐galactosidase activity can be assumed to be correlated with *lacZ* gene expression, and because the *lacZ* gene was positioned at the end of the gene cluster, the reporter activity is indicative of the transcription level of the gene cluster. Quantification of reporter activity thus enables selection of clones with different gene cluster expression levels which may increase the chance to identify clones in which the target pathway could be ‘transplanted’ successfully. Accordingly, cultivate *Pseudomonas* clones under chosen conditions for metabolite production for quantification of *β*‐galactosidase activity and subject samples to an ONPG (*o*‐nitrophenyl *β*‐d‐galactopyranoside) assay. The ONPG assay is based on the *β*‐galactosidase‐mediated cleavage of the substrate releasing *o*‐nitrophenol which can be photometrically detected, and the calculation of Miller units as a measure of enzyme activity (Miller, 1972). To conduct a modified Miller assay, first measure cell densities (OD_600 nm_) and subject samples of cell cultures to the ONPG assay: mix 10 μl cell culture samples with 390 μl diluted Z‐buffer to generate a ‘sample solution’ with a total volume of 400 μl (sample and buffer volumes may be adapted if necessary, i.e. if activities are too high or too low). Add 25 μl chloroform and 25 μl Z‐buffer, and mix well. After incubation at 30°C for 3 min, add 400 μl of ONPG substrate solution, followed by a 2‐min incubation, before addition of 400 μl stop solution. After removal of cell debris by centrifugation (22°C, 1 min, 21 000 *g*), measure *o*‐nitrophenol absorption of samples at 420 nm [e.g. in the microplate reader TECAN Infinite^®^ M1000 PRO (Tecan Deutschland GmbH, Crailsheim, Germany)]. Based on the data, calculate Miller units, taking into account the individual cell densities of the cultures, the employed sample volumes relative to the total assay volume, the assay incubation time, and the background signal measured in control samples without cells. All ONPG assay solutions and the details of Miller unit calculation are listed in the Table S3. Categorize strains based on results and proceed with further suitable analysis of metabolite production using representatives of different expression categories.

## An example of yTREX application: Generation and *lacZ*‐based categorization of PCA‐producing *P. putida* strains

*Background*: The yTREX tool is designed to enable the easy generation of bacterial strains expressing biosynthetic genes, which can be achieved by random transposon Tn5‐based chromosomal integration downstream of a suitable promoter. This tool further facilitates the identification of gene cluster expressing strains within a library of non‐expressing clones by simple co‐integration of a reporter gene (e.g. *lacZ*) and the respective readout (see sections Design of the expression construct–yTREX gene cluster transfer, transposon integration & implementation of gene expression). To investigate if the level of reporter activity could be used to estimate levels of the biosynthetic product, *β*‐galactosidase‐mediated indication of recombinant phenazine production in *P. putida* KT2440 was chosen as an example. The chorismic acid‐derived group of phenazine compounds displays antibacterial and antifungal properties (Simionato *et al*., 2017; Zhang *et al*., 2017; Zhu *et al*., 2017) and *P. putida* KT2440, a safe and widely established representative workhorse of the *Pseudomonas* clade, is able to withstand production of the compound (Domröse *et al*., 2017).

As described in a previous study (Domröse *et al*., 2017), we have integrated the phenazine‐1‐carboxylic acid (PCA) biosynthesis‐encoding genes *phzA1B1C1D1E1F1G1* from *P. aeruginosa*, together with the promotor‐less *β‐*galactosidase‐encoding *lacZ* gene from *E. coli* in the yTREX vector (employing protocols described in sections Design of the expression construct–Generation of gene cluster DNA fragments & yeast assembly cloning in the yTREX vector, see detailed scheme and description of the procedure in Fig. S1). This construct was used for random Tn5‐based gene cluster integration into the *P. putida* KT2440 chromosome, and we have previously demonstrated that the reporter‐based agar plate‐based blue/white screening (as described in section yTREX gene cluster transfer, transposon integration & implementation of gene expression) allowed distinguishing between expressing and non‐expressing clones: while white clones showed no production of PCA, blue‐coloured clones accumulated the compound. In order to analyse if the levels of reporter activity could be used to indicate metabolite production levels, we here comparatively quantified *β*‐galactosidase activities of six clones, which showed target gene transcription according to the blue/white assay including previously described strains PCA1, PCA2, and PCA8 (Domröse *et al*., 2017), *via* an ONPG assay. PCA production levels of the same clones were determined by HPLC‐PDA analysis and compared to the corresponding *β‐*galactosidase activities.

**Figure 4:**
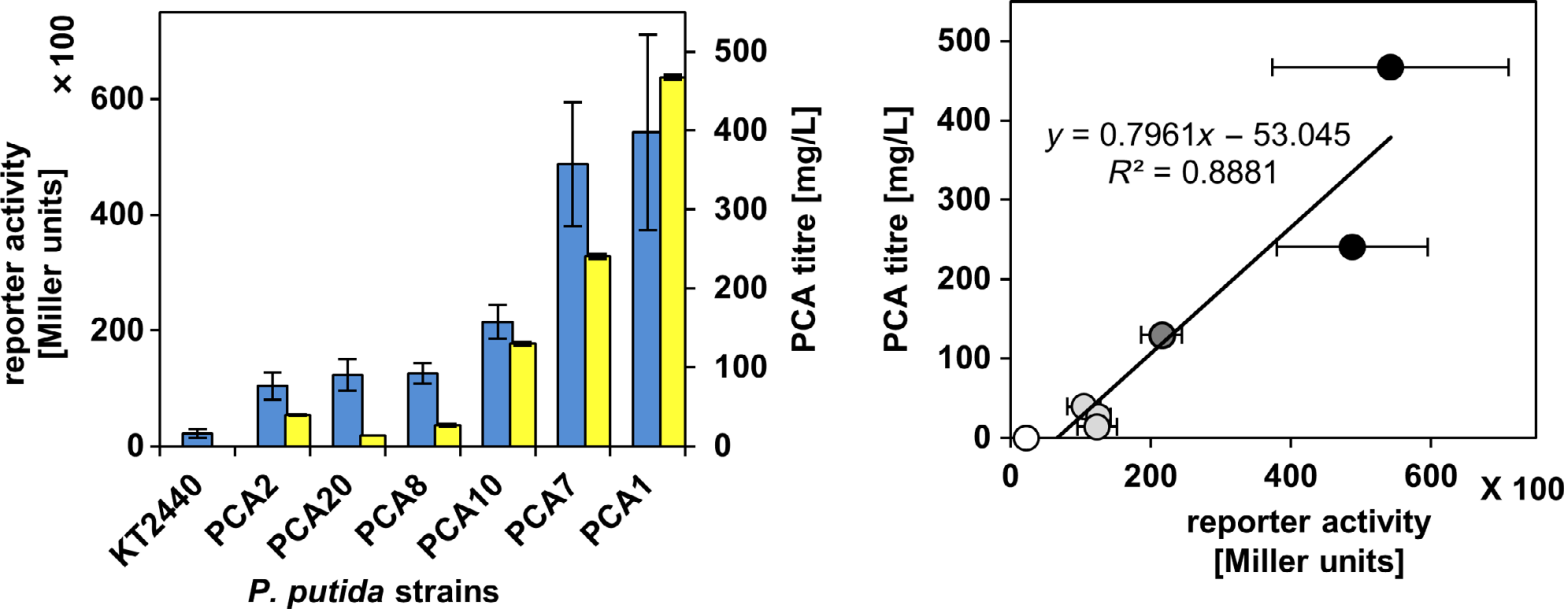
Correlation of *β*‐galactosidase reporter activity and PCA production of *P. putida* strains carrying the yTREX‐phzA‐G‐lacZ transposon. Left panel: Blue bars represent *β*‐galactosidase reporter activities of six *P. putida* KT2440 clones carrying the recombinant yTREX‐phzA‐G‐lacZ transposon [left axis, Milller units]. Yellow bars represent PCA titres in the *P. putida* cultures [right axis, mg/l. Names of *P. putida* strains are indicated. Both data sets represent mean values of triplicate measurements with the respective standard deviation. Right panel: Reporter activity and PCA titres were plotted against each other. Strains were grouped into categories (see text) showing no, relatively low, intermediate and high reporter activity and production of PCA (from white to black fillings). All data points were included in a linear fit (black line with respective equation).

*Methodology*: Pre‐cultures were inoculated with single colonies of individual clones of the chosen *P. putida* strains in 1 ml liquid LB medium in Round Well Plates (m2p‐labs GmbH, Baesweiler, Germany) and cultivated for 16 h (30°C, shaking at 900 rpm). Subsequently, aliquots of the pre‐cultures were used to inoculate test cultures (starting OD_600 nm_ = 0.05) in TB medium (12 g/l casein, 24 g/l yeast extract, 9.4 g/l K_2_HPO_4_, 2.2 g/l KH_2_PO_4_, 4 ml/l glycerol; Carl‐Roth, Karlsruhe, Germany) in Round Well Plates (m2p‐labs GmbH, Baesweiler, Germany), which were cultivated for 24 h (30°C, shaking at 900 rpm). For determination of *β*‐galactosidase activities, cell densities (OD_600 nm_) were measured and 10 μl samples of each culture were subjected to the ONPG assay (as described in section yTREX gene cluster transfer, transposon integration & implementation of gene expression). In parallel, 750 μl samples of the same *P. putida* cultures were subjected to PCA extraction and analysis as previously described in detail (Domröse *et al*., 2017). In brief, PCA was extracted twice with 0.5 ml ethyl acetate from cell‐free culture supernatant after acidification by addition of 80 μl of 6 M HCl. Samples were then dried and re‐suspended in 1–10 ml ethanol as appropriate for completely solving the extract. Aliquots (10 μl) of these solutions were subjected to HPLC‐PDA measurements, using an authentic reference for correlating the peak area signal (in a chromatogram recorded at 366 nm) to the compound's concentration in the sample. Based on that, product titres (in mg/l culture) were calculated taking into account the total sample volume of which an aliquot was used for analysis, and the volume of bacterial culture that was used for extraction. The results are shown in Fig. 4.

*Results*: While the wild‐type strain KT2440 exhibited a background activity of ~2200 Miller units, *β*‐galactosidase activities of strains PCA1, PCA2, PCA7, PCA8, PCA10 and PCA20, which carry the recombinant yTREX‐phzA‐G‐lacZ transposon at random chromosomal integration sites, ranged from ~10 400 to ~54 200. Data were sorted in ascending order (Fig. 4), enabling the following categorization: Three strains showed comparatively low *β*‐galactosidase activity (PCA2/PCA20/PCA8; ~11 500 Miller units), one strain showed intermediate activity (PCA10; ~21 500 Miller units) and two high activity (PCA7/PCA1; ~51 000 Miller units). Remarkably, investigation of phenazine production showed that the same three strains produced comparatively low amounts of PCA (PCA2/PCA20/PCA8; 14–39 mg/l), and the other three strains showed significantly higher production in the same order (PCA10/PCA7/PCA1; 129–467 mg/l). Data were thus plotted against each other and included in a linear fit to describe the correlation of reporter activity with PCA production. Clearly, the obtained fit lacks further data points for statistical backing of this evaluation; however, the plot indicates a clear trend which allowed categorizing strains as low, intermediate or rather high‐level producers based on the measured reporter activity. Although the two data sets are indeed expected to be both dependent on the transcription strength from the same promoter, a strict linear correlation cannot generally be expected as the reporter activity and compound production can be differentially influenced by numerous other variables: while the *β*‐galactosidase reporter activity is long‐known for providing a rather robust readout under diverse conditions, the accumulation of a biosynthetic product is influenced by multiple factors, for example active protein formation, metabolic fluxes, toxicity‐associated issues as well as diffusion or secretion processes. Effects on the level of mRNA translation can, in some cases, even lead to an inverse correlation of transcript levels and the levels of active protein (Song *et al*., 2016). Finally, the local chromosomal context at an insertion site might in addition differentially influence transcript stabilities of the biosynthetic genes vs. the reporter gene by enhancing or decreasing degradation of 3′‐mRNA termini (Belasco, 2010; Deutscher, 2015). However, in the here presented proof of concept experiment, a correlation between *β*‐galactosidase activity and PCA formation was observed, and the method is applicable not only to identify expressing clones in a high‐throughput manner but also to categorize strains as low, intermediate or high‐level PCA producers.

## Discussion

The facile application of the yTREX tool for Tn5‐based random genomic integration can enable the effective expression of biosynthetic gene clusters in *P. putida*. Despite being a multi‐step procedure, the yTREX protocol is relatively easy to implement: all components, that is protocols, sequence information and yTREX DNA, are readily available. Each sub‐step of the application protocol is rooted in widely established, robust methods and can be conducted in laboratories with standard equipment.

When working with large gene clusters, the cloning step is certainly still a bottleneck. Although we have also encountered difficulties with apparently challenging target DNA, the yeast cloning protocol is in our hands a reliable method for gene cluster assembly. Alternative methods for the conduction of cloning steps may be type IIS restriction endonuclease‐based approaches like Golden Gate Cloning or MIDAS, Gibson assembly or phage recombinase‐based cloning (Engler *et al*., 2008; Gibson, 2011; Fu *et al*., 2012; Van Dolleweerd *et al*., 2018).

The yeast recombineering method facilitates the implementation of different design concepts for gene cluster expression. Notably, transposon Tn5‐based gene cluster integration creates multiple clones with different transposon insertion sites and can readily install various gene expression levels of the entire gene cluster if it exhibits unidirectional gene orientation. The addition of the commonly employed *lacZ* reporter gene to the 3′‐end of the gene cluster enables the identification of gene cluster transcribing clones within the transposon library and renders application very handy. It moreover allows quantifying expression levels. Since the levels of transcription and translation can have a positive or negative influence on the metabolic flux and biosynthetic product yields in heterologous pathway establishment, the determination of expression levels can help to optimize production. Especially for unknown pathways and products, it may be useful to first determine the reporter activity as a measure of gene cluster transcription strength to select a limited number of strains before elaborate investigation of biosynthetic success.

It should be noted that highly sophisticated tools are available to achieve an accurate, marker‐less and efficient chromosomal integration of biosynthetic gene clusters in the *P. putida* chromosome, for example *via* phage recombinases (Choi *et al*., 2018). At the same time, a broad variety of calibrated constitutive and inducible promoters has been established (Zobel *et al*., 2015; Calero *et al*., 2016; Elmore *et al*., 2017; Nikel and de Lorenzo, 2018). Their combined use can likewise allow the installation of different defined and even gradually tuneable expression levels in the host. However, as opposed to the targeted gene integration and use of different promoters with known strength, the Tn5‐based procedure does not require any previous knowledge on the functionality of promoter systems for a specific host. In addition, the cloning of a single expression construct is sufficient to achieve expression at various levels, even in different hosts. Furthermore, it allows exploiting the entire chromosomal space to identify novel promoters and sites in the bacterial host chromosome that are suitable for the expression of respective gene clusters (Domröse *et al*., 2015; A. Domröse and A. Loeschcke, unpublished). Therefore, both the site‐specific and random gene cluster integration offer very specific benefits (and drawbacks) and are thus complementary to one another useful to address different types of scientific questions. We are currently testing methods for targeted chromosomal integration and alternative expression concepts to further expand the yTREX system in a versatile toolbox concept.

In summary, the yTREX protocol allows the fast assessment of a host's suitability for the production of a compound of interest. We have focused on the host *P. putida* KT2440 (Domröse *et al*., 2017) but the yTREX tool is likewise applicable for other bacteria (Loeschcke *et al*., 2013) including any *Pseudomonas* species. This can not only enable the production of natural metabolites, but also contribute to the discovery and investigation of novel pathways or the combinatorial biosynthesis of new compounds.

## Conflict of interest

None declared.

## Supporting information


**Fig. S1.** Assembly scheme of yTREX‐phzA‐GlacZ.
**Table S1.** yTREX vector scheme and DNA sequence.
**Table S2.** Employed *β*‐galactosidase‐encoding *lacZ* gene sequence.
**Table S3.** Assay solutions and calculations for determining Miller units.
**Appendix S1.** Yeast transformation based on previously established methodology.
