# Supplementary material for: Protocols for yTREX/Tn5‐based gene cluster expression in Pseudomonas putida
Source: Microb Biotechnol. 2019 Jun 4;13(1):250–62. doi: 10.1111/1751-7915.13402 (PMC6922528; doi:10.1111/1751-7915.13402)
Supplement: Supplementary file 1 — Fig. S1. Assembly scheme of yTREX‐phzA‐GlacZ. Table S1. yTREX vector scheme and DNA sequence. Table S2. Employed β‐galactosidase‐encoding lacZ gene sequence. Table S3. Assay solutions and calculations for determining Miller units. Appendix S1. Yeast transformation based on previously established methodology. [file MBT2-13-250-s001.pdf]

Supporting information

Protocols for yTREX/Tn5-based gene cluster expression in *Pseudomonas putida*

Robin Weihmann[1], Andreas Domröse[1], Thomas Drepper[1], Karl-Erich Jaeger[1], Anita Loeschcke[1]*

[1]Institute of Molecular Enzyme Technology, Heinrich-Heine-Universität Düsseldorf, Forschungszentrum Jülich, Jülich, Germany

*corresponding author:
Anita Loeschcke, a.loeschcke@fz-juelich.de

Content **Page**

Figure S1. Assembly scheme of yTREX-phzA-GlacZ 2

Table S1. yTREX vector scheme and DNA sequence 4

Table S2. Employed β-galactosidase-encoding *lacZ* gene sequence 8

Table S3. Assay solutions and calculations for determining Miller units 9

Appendix S1. Yeast transformation based on previously established methodology 10

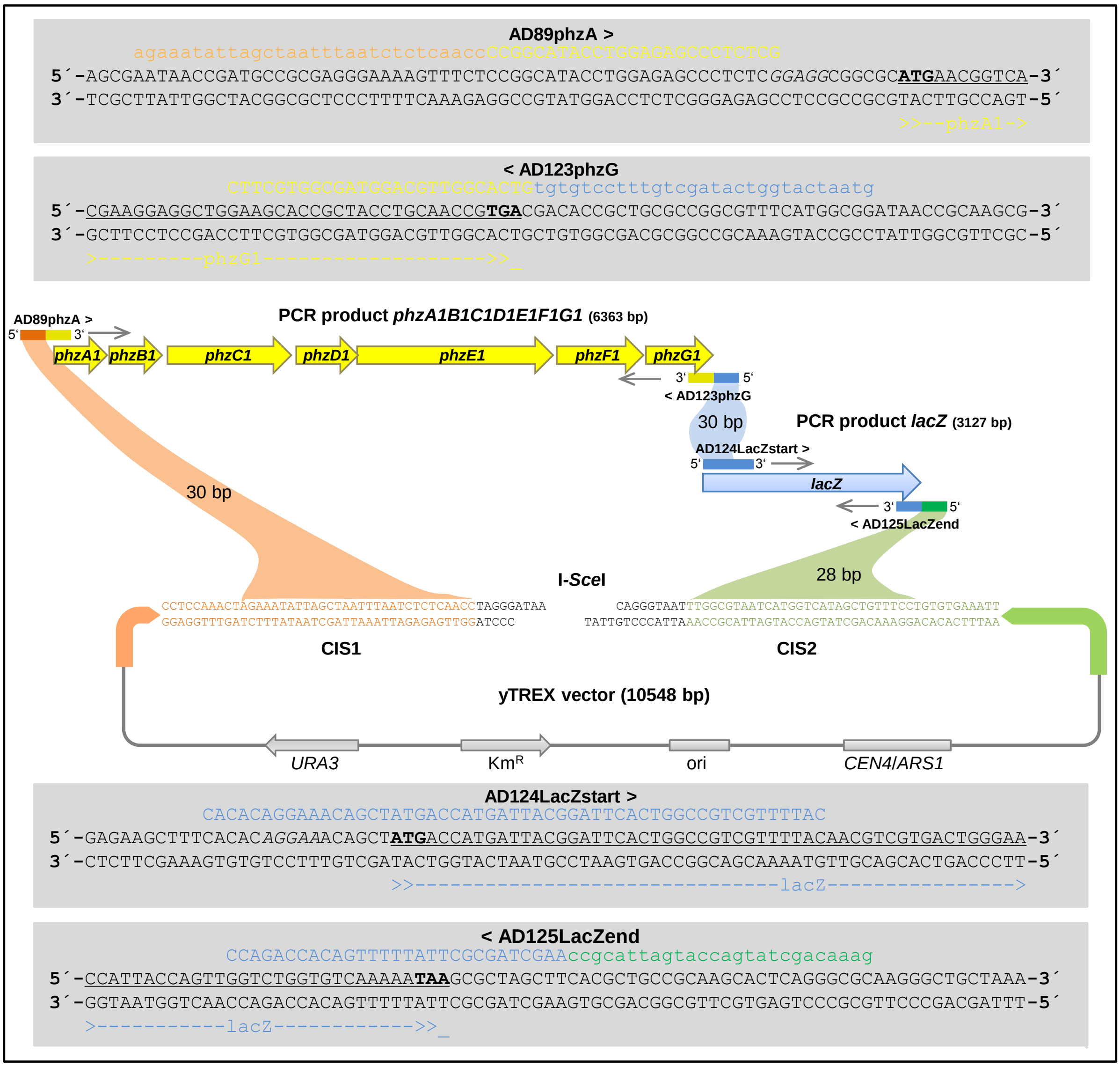

Figure S1. Assembly scheme of yTREX-phzA-G-lacZ.

The yTREX vector is depicted schematically after linearisation using I-*Sce*I. The PCR fragments of *phz* genes (*P. aeruginosa*) and *lacZ* (*E. coli*) are shown with primers adding homologous overlapping regions between the fragments and to the CIS sequences of the vector exhibiting a typical length (28 and 30 bp). Depiction is not drawn to scale. Primer sequences (colour-coded as in the graphic) and their positions on template sequences (shown as double strands) are specified in grey boxes (binding sequences of primers are shown in capital letters, non-binding elongations in small caps; coding gene sequences in the templates are underlined; start/stop codons are marked in bold; purine-rich regions of (putative) RBS sequences are highlighted in italic font). Cloning and application of the vector yTREX-phzA-G-lacZ has been described in the original publication: Domröse *et al*., 2017; doi: 10.1016/j.synbio.2017.11.001. The conceptualisation and vector assembly (see sections 3.1 and 3.2 of this manuscript) were in detail conducted as follows:

<u>1 Conceptualisation of the expression cassette:</u>

As expression mode of the PCA biosynthesis genes, we chose to rely on a chromosomal *P. putida* promoter. Therefore, the assembled gene cluster needed to exhibit unidirectional gene organisation without transcription termination sites. As the *phzA1B1C1D1E1F1G1* genes naturally occur as a single transcriptional unit in *P. aeruginosa* PAO1, no re-arrangements of the biosynthetic genes was necessary. In addition, we aimed for the detection of gene cluster transcription after transposon integration *via* the *lacZ* reporter gene, which thus needed to be included downstream of the gene cluster.

2 Primer design for amplifying the biosynthetic genes and the reporter gene:

The biosynthetic *phzA1B1C1D1E1F1G1* genes span 6.4 kb and were thus PCR amplified in a single reaction without subdivision into smaller sections. As template, we used vector pUC18-pyo (S. Thies, unpublished; carrying the *phzA1B1C1D1E1F1G1* genes of *P. aeruginosa* PAO1). The forward primer was designed to contain a 25 bp 3'-binding sequence (Tm 73 °C) complementary to the 5'-UTR of the first gene *phzA1* to include the RBS but exclude the native promoter of the *phz* genes. A 30 bp elongation was added to the 5'-end of the forward primer as a homology arm complementary to the CIS1 of the L-yTREX. The overall forward primer Tm was 77 °C. The reverse primer was designed to contain a 25 bp 3'-binding sequence (Tm 73 °C) to the 3'-end of the coding sequence of the *phzG1* gene including the respective stop codon (but no further sequences that could include a transcription termination) and a 5'-elongation (30 bp) complementary to the *lacZ* PCR product (specifically the 5'-UTR including the RBS and start of the coding sequence of *lacZ*). The overall reverse primer Tm was 80 °C.

Forward primer AD89phzA:

5'-AGAAATATTAGCTAATTTAATCTCTCAACCCCGGCATACCTGGAGAGCCCTCTCG-3'

Reverse primer AD123phzG:

5'-GTAATCATGGT**CAT**AGCTGTTTCCTGTGTG**TCA**CGGTTGCAGGTAGCGGTGCTTC-3'

To enable transcription reporter function, the *lacZ* gene was amplified including its native 5'-UTR including the RBS but no promoter. As template, we used pRcExpII2-YF1-FixJ-PFixK2lacZ (A. Loeschcke, unpublished; carrying the *lacZ* gene of *E. coli*, see **supporting Table S1**). The forward primer was designed to fully bind (53 bp, Tm 78 °C) to the template 5'-UTR and start of the coding sequence of *lacZ*. We omitted a 5'-elongation on this primer and did not add a homology arm with a complementary sequence to the PCR product containing the *phz* genes, because this enabled us to make use of the same *lacZ* PCR product for the assembly of multiple constructs with different biosynthetic genes (not described in this manuscript). The reverse primer was designed to contain a 27 bp 3'-binding sequence (Tm 68 °C) to the 3'-end of *lacZ* including the respective stop codon and a 5'-elongation (28 bp) complementary to CIS2 of the R-yTREX. The overall reverse primer Tm was 78 °C.

Forward primer AD124LacZstart:

5'-CACAC*AGGAAA*CAGCT**ATG**ACCATGATTACGGATTCACTGGCCGTCGTTTTAC-3'

Reverse primer AD125LacZend:

5'-GAAACAGCTATGACCATGATTACGCCAAGCTAGCGC**TTA**TTTTTGACACCAGACC-3'

All primer melting temperatures were determined using CloneManager (Sci Ed Software). Binding and elongation sequences of primers are colour coded as in the **Figure S1**, coding sequences are underlined and start or stop codons highlighted in bold font, while purine-rich regions with putative RBS sequences are marked in italic font.

3 Preparation of DNA fragments for vector assembly *via* restriction hydrolysis/PCR:

The yTREX vector was linearized using I-*Sce*I (as described under section 3.2 of this manuscript).

The *phz* genes and the *lacZ* gene were PCR-amplified. For the *phzA1B1C1D1E1F1G1* fragment, the PCR mix with a total volume of 50 µL was composed of 1 µL template DNA (850 ng/µL), 0.5 µL primers (10 pmol/µL each), 1 µL dNTPs (10 mM), 5 µL DMSO, 0.5 µL Phusion polymerase and 10 µL 5x Phusion GC buffer (both Thermo Fisher Scientific GmbH, Walkham, USA) as well as 31.5 µL nuclease-free water. The reaction was performed according to the manufacturer's manual, choosing a two-step protocol with combined annealing and elongation phases (2.25 min) at 72 °C for 35 cycles. For the amplification of *lacZ*, the PCR mix was composed analogously. The reaction was likewise performed with a two-step protocol using combined annealing and elongation phases (1 min) at 72 °C for 35 cycles. PCR products were not isolated by agarose gel electrophoresis and spin column purification in this case, because no unspecific products were obtained and no templates that are replicative plasmids in yeast (which could lead to growth of false-positive clones) were used.

4 Assembly of the vector by yeast recombineering:

To obtain the vector yTREX-phzA-G-lacZ, the yTREX vector fragment was assembled with the two insert fragments (*phzA1B1C1D1E1F1G1* and *lacZ*) by concerted transformation in *S. cerevisiae*, following the generally applicable steps described under section 3.2 of this manuscript. After plasmid

amplification using *E. coli*, isolated putative yTREX-phzA-G-lacZ vector DNA was analysed by restriction hydrolysis and agarose gel electrophoresis which resulted in the expected band pattern. In addition, correct sequences in the regions where homologous recombination took place were verified by Sanger sequencing.

Supporting Table S1. yTREX vector scheme and DNA sequence. The relevant features of the yTREX vector (Domröse *et al.*, 2017; doi: 10.1016/j.synbio.2017.11.001) are colour coded, in accordance with the provided schematic representation of the yTREX cassettes. The sequence (NCBI GenBank MK416190) is here given in GenBank format and can be readily imported into cloning software like CloneManager (from Sci Ed Software). The CIS1 and CIS2 sequences, which were designed to match the sequences up- and downstream of prodigiosin biosynthetic genes in plasmid pPIG (Loeschcke et al., 2013; doi: 10.1021/sb3000657) to enable their integration in the yTREX vector for our first application of this tool, can be used as target sequences for homologous recombination-based integration of a given gene cluster of interest.

Schematic representation of the yTREX vector

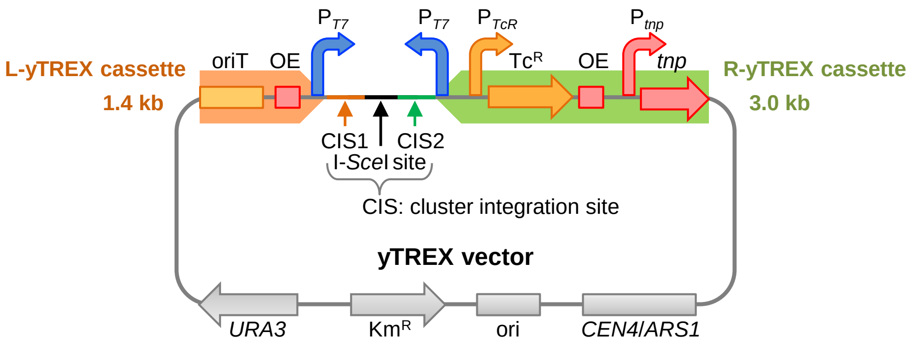

yTREX vector sequence and feature annotations (GenBank format)

```
LOCUS       yTREX_vector          10548 bp    DNA     circular SYN 21-JAN-2019
DEFINITION  yTREX vector (Domröse et al., 2017; doi:
            10.1016/j.synbio.2017.11.001)
COMMENT     SECID/File created by Clone Manager, Scientific & Educational Software
FEATURES             Location/Qualifiers
     misc_feature    41..1371
                     /gene="L-yTREX"
                     /product="L-yTREX cassette from SpeI site to T7 promotor."
     misc_feature    complement(53..1244)
                     /gene="oriT"
                     /product="oriT mobilisation sequence, from pBBR1; Antoine
                     & Locht, Mol Microbiol. 1992, 6(13):1785-99."
     misc_feature    1245..1263
                     /gene="OE"
                     /product="outside end of transposon Tn5 (L-yTREX);
                     Reznikoff, Annu Rev Genet. 2008;42:269-86."
     misc_feature    1350..1371
                     /gene="P T7"
                     /product="bacteriophage T7 promoter (L-yTREX); Dunn &
                     Studier, J Mol Biol. 1983, 166(4):477-535."
     misc_feature    1372..1411
                     /gene="CIS1"
                     /product="cluster integration site: sequence for
                     homologous recombination with inserts (L-yTREX)"
     misc_feature    1412..1429
                     /gene="I-SceI"
                     /product="recognition site of homing endonucleaase
                     I-SceI."
     misc_feature    1430..1469
                     /gene="CIS2"
                     /product="cluster integration site: sequence for
                     homologous recombination with inserts (R-yTREX)"
     misc_feature    complement(1470..1491)
                     /gene="P T7"
                     /product="bacteriophage T7 promoter (R-yTREX); Dunn &
                     Studier, J Mol Biol. 1983, 166(4):477-535."
     misc_feature    complement(1470..4385)
                     /gene="R-yTREX"
                     /product="R-yTREX cassette from SpeI site to T7 promoter."
     misc_feature    1532..1597
                     /gene="P TcR"
                     /product="TcR promoter region, from E. coli plasmid
                     pBR322; Harley et al, Nucleic Acids Res. 1988,
                     16(15):7269-85."
     CDS             1620..2810
                     /gene="TcR"
                     /product="TcR, from E. coli plasmid pBR322; Sutcliffe,
                     Cold Spring Harb Symp Quant Biol. 1979;43 Pt 1:77-90."
                     /translation="MKSNNALIVILGTVTLDAVGIGLVMPVLPGLLRDIVHSDSIASH
                     YGVLLALYALMQFLCAPVLGALSDRFGRRPVLLASLLGATIDYAIMATTPVLWILYAG
                     RIVAGITGATGAVAGAYIADITDGEDRARHFGLMSACFGVGMVAGPVAGGLLGAISLH
                     APFLAAAVLNGLNLLLGCFLMQESHKGERRPMPLRAFNPVSSFRWARGMTIVAALMTV
                     FFIMQLVGQVPAALWVIFGEDRFRWSATMIGLSLAVFGILHALAQAFVTGPATKRFGE
```

```
                 KQAIIAGMAADALGYVLLAFATRGWMAFPIMILLASGGIGMPALQAMLSRQVDDDHQG
                 QLQGSLAALTSLTSIIGPLIVTAIYAASASTWNGLAWIVGAALYLVCLPALRRGAWSR
                 ATST"
     misc_feature    complement(2851..2869)
                 /gene="OE"
                 /product="outside end of transposon Tn5 (R-yTREX);
                 Reznikoff, Annu Rev Genet. 2008;42:269-86."
     misc_feature    2880..2910
                 /gene="P tnp"
                 /product="Transposon Tn5 P tnp promoter sequence (-35 to
                 -10); Yin et al, J Mol Biol. 1988, 99(1):35-45."
     CDS             2943..4373
                 /gene="tnp"
                 /product="Transposon Tn5 transposase gene; Goryshin &
                 Reznikoff, J Biol Chem. 1998, 273(13):7367-74."
                 /translation="MITSALHRAADWAKSVFSSAALGDPRRTARLVNVAAQLAKYSGK
                 SITISSEGSEAMQEGAYRFIRNPNVSAEAIRKAGAMQTVKLAQEFPELLAIEDTTSLS
                 YRHQVAEELGKLGSIQDKSRGWWVHSVLLLEATTFRTVGLLHQEWWMRPDDPADADEK
                 ESGKWLAAAATSRLRMGSMMSNVIAVCDREADIHAYLQDKLAHNERFVVRSKHPRKDV
                 ESGLYLYDHLKNQPELGGYQISIPQKGVVDKRGKRKNRPARKASLSLRSGRITLKQGN
                 ITLNAVLAEEINPPKGETPLKWLLLTSEPVESLAQALRVIDIYTHRWRIEEFHKAWKT
                 GAGAERQRMEEPDNLERMVSILSFVAVRLLQLRESFTLPQALRAQGLLKEAEHVESQS
                 AETVLTPDECQLLGYLDKGKRKRKEKAGSLQWAYMAIARLGGFMDSKRTGIASWGALW
                 EGWEALQSKLDGFLAAKDLMAQGIKI"
     misc_feature    4885..4995
                 /gene="Cen4"
                 /product="CEN4 of S.cerevisiae Chr IV (Saccharomyces
                 Genome Database)."
     misc_feature    5527..6271
                 /gene="ARS1"
                 /product="ARS1 of yCP50; GenBank: X70276.1."
     misc_feature    6741..7329
                 /gene="pMB1 ori"
                 /product="origin of replication from E. coli plasmid
                 pBR322 (Sutcliffe, Cold Spring Harb Symp Quant Biol.
                 1979;43 Pt 1:77-90); annotated as New England Biolabs
                 (2007)."
     CDS             complement(7508..8302)
                 /gene="KmR"
                 /product="aphII gene, aminoglycoside
                 3'-phosphotransferase, kanamycin resistance gene;
                 Pridmore, Gene. 1987;56(2-3):309-12."
                 /translation="MIEQDGLHAGSPAAWVERLFGYDWAQQTIGCSDAAVFRLSAQGR
                 PVLFVKTDLSGALNELQDEAARLSWLATTGVPCAAVLDVVTEAGRDWLLLGEVPGQDL
                 LSSHLAPAEKVSIMADAMRRLHTLDPATCPFDHQAKHRIERARTRMEAGLVDQDDLDE
                 EHQGLAPAELFARLKARMPDGEDLVVTHGDACLPNIMVENGRFSGFIDCGRLGVADRY
                 QDIALATRDIAEELGGEWADRFLVLYGIAAPDSQRIAFYRLLDEFF"
     misc_feature    complement(8392..8440)
                 /gene="P KmR"
                 /product="aphII promoter sequence, from transposon Tn5,
                 GenBank: U00004.1."
     misc_feature    8740..8955
                 /gene="P URA3"
                 /product="URA3 promoter region; Roy et al, Mol Cell Biol.
                 1990, 10(10):5257-70."
     CDS             8956..9759
                 /gene="URA3"
                 /product="ORF frame 2"
                 /translation="MSKATYKERAATHPSPVAAKLFNIMHEKQTNLCASLDVRTTKEL
                 LELVEALGPKICLLKTHVDILTDFSMEGTVKPLKALSAKYNFLLFEDRKFADIGNTVK
                 LQYSAGVYRIAEWADITNAHGVVGPGIVSGLKQAAEEVTKEPRGLLMLAELSCKGSLS
                 TGEYTKGTVDIAKSDKDFVIGFIAQRDMGGRDEGYDWLIMTPGVGLDDKGDALGQQYR
                 TVDDVVSTGSDIIIVGRGLFAKGRDAKVEGERYRKAGWEAYLRRCGQQN"
ORIGIN
        1 gccattctca tgaagaatat cttgaattta ttgtcatatt actagtcccg ggtccacata
       61 tccacgggct ggcaagggag cgcagcgacc gcgcagggcg aagcccggag agcaagcccg
      121 tagggcgccg cagccgccgt aggcggtcac gactttgcga agcaaagtct agtgagtata
      181 ctcaagcatt gagtggcccg ccggaggcac cgccttgcgc tgcccccgtc gagccggttg
      241 gacaccaaaa gggaggggca ggcatggcgg catacgcgat catgcgatgc aagaagctgg
      301 cgaaaatggg caacgtggcg gccagtctca agcacgccta ccgcgagcgc gagacgccca
      361 acgctgacgc cagcaggacg ccagagaacg agcactgggc ggccagcagc accgatgaag
      421 cgatgggccg actgcgcgag ttgctgccag agaagcggcg caaggacgct gtgttggcgg
      481 tcgagtacgt catgacggcc agcccggaat ggtggaagtc ggccagccaa gaacagcagg
      541 cggcgttctt cgagaaggcg cacaagtggc tggcggacaa gtacggggcg gatcgcatcg
      601 tgacggccag catccaccgt gacgaaacca gcccgcacat gaccgcgttc gtggtgccgc
      661 tgacgcagga cggcaggctg tcggccaagg agttcatcgg caacaaagcg cagatgaccc
      721 gcgaccagac cacgtttgcg gccgctgtgg ccgatctagg gctgcaacgg ggcatcgagg
      781 gcagcaaggc acgtcacacg cgcattcagg cgttctacga ggccctggag cggccaccag
      841 tgggccacgt caccatcagc ccgcaagcgg tcgagccacg cgcctatgca ccgcagggat
      901 tggccgaaaa gctgggaatc tcaaagcgcg ttgagacgcc ggaagccgtg gccgaccggc
      961 tgacaaaagc ggttcggcag gggtatgagc ctgccctaca ggccgccgca ggagcgcgtg
     1021 agatgcgcaa gaaggccgat caagcccaag agacggcccg agaccttcgg gagcgcctga
     1081 agcccgttct ggacgccctg gggccgttga atcgggatat gcaggccaag gccgccgcga
     1141 tcatcaaggc cgtgggcgaa aagctgctga cggaacagcg ggaagtccag cgccagaaac
     1201 aggcccagcg ccagcaggaa cgcgggcgcg cacatttccc cgaactgact cttatacaca
     1261 agtgttcttt cctgcgttat cccctgattc tgtggataac cgtgaattcg gtttcccgac
     1321 tggaaagcgg gcagtgagcg caacgcaata attaatacga ctcactatag gcctccaaac
     1381 tagaaatatt agctaattta atctctcaac ctagggataa cagggtaatt tggcgtaatc
```

```
1441 atggtcatag ctgtttcctg tgtgaaattc ctatagtgag tcgtattaat taggttgggc
1501 ttcggaatcg ttttccggga cgccggctgg agaattctca tgtttgacag cttatcatcg
1561 ataagcttta atgcggtagt ttatcacagt taaattgcta acgcagtcag gcaccgtgta
1621 tgaaatctaa caatgcgctc atcgtcatcc tcggcaccgt caccctggat gctgtaggca
1681 taggcttggt tatgccggta ctgccgggcc tcttgcggga tatcgtccat tccgacagca
1741 tcgccagtca ctatggcgtg ctgctagcgc tatatgcgtt gatgcaattt ctatgcgcac
1801 ccgttctcgg agcactgtcc gaccgctttg gccgccgccc agtcctgctc gcttcgctac
1861 ttggagccac tatcgactac gcgatcatgg cgaccacacc cgtcctgtgg atcctctacg
1921 ccggacgcat cgtggccggc atcaccggcg ccacaggtgc ggttgctggc gcctatatcg
1981 ccgacatcac cgatggggaa gatcgggctc gccacttcgg gctcatgagc gcttgtttcg
2041 gcgtgggtat ggtggcaggc cccgtggccg ggggactgtt gggcgccatc tccttgcatg
2101 caccattcct tgcggcggcg gtgctcaacg gcctcaacct actactgggc tgcttcctaa
2161 tgcaggagtc gcataaggga gagcgtcgac cgatgccctt gagagccttc aacccagtca
2221 gctccttccg gtgggcgcgg ggcatgacta tcgtcgccgc acttatgact gtcttcttta
2281 tcatgcaact cgtaggacag gtgccggcag cgctctgggt cattttcggc gaggaccgct
2341 ttcgctggag cgcgacgatg atcggcctgt cgcttgcggt attcggaatc ttgcacgccc
2401 tcgctcaagc cttcgtcact ggtcccgcca ccaaacgttt cggcgagaag caggccatta
2461 tcgccggcat ggcggccgac gcgctgggct acgtcttgct ggcgttcgcg acgcgaggct
2521 ggatggcctt ccccattatg attcttctcg cttccggcgg catcgggatg cccgcgttgc
2581 aggccatgct gtccaggcag gtagatgacg accatcaggg acagcttcaa ggatcgctcg
2641 cggctcttac cagcctaact tcgatcattg gaccgctgat cgtcacggcg atttatgccg
2701 cctcggcgag cacatggaac gggttggcat ggattgtagg cgccgcccta taccttgtct
2761 gcctccccgc gttgcgtcgc ggtgcatgga gccgggccac ctcgacctga agagcgccca
2821 atacgcaaac cgcctctccc cgcgcgttgg acttgtgtat aagagtcaga gcgtcctgaa
2881 cggaaccttt cccgttttcc aggatctgat cttccatgtg acctcctaac atggtaacgt
2941 tcatgataac ttctgctctt catcgtgcgg ccgactgggc taaatctgtg ttctcttcgg
3001 cggcgctggg tgatcctcgc cgtactgccc gcttggttaa cgtcgccgcc caattggcaa
3061 aatattctgg taaatcaata accatctcat cagagggtag tgaagccatg caggaaggcg
3121 cttaccgatt tatccgcaat cccaacgttt ctgccgaggc gatcagaaag gctggcgcca
3181 tgcaaacagt caagttggct caggagtttc ccgaactgct ggccattgag gacaccacct
3241 ctttgagtta tcgccaccag gtcgccgaag agcttggcaa gctgggctct attcaggata
3301 aatcccgcgg atggtgggtt cactccgttc tcttgctcga ggccaccaca ttccgcaccg
3361 taggattact gcatcaggag tggtggatgc gcccggatga ccctgccgat gcggatgaaa
3421 aggagagtgg caaatggctg gcagcggccg caactagccg gttacgcatg ggcagcatga
3481 tgagcaacgt gattgcggtc tgtgaccgcg aagccgatat tcatgcttat ctgcaggaca
3541 aactggcgca taacgagcgc ttcgtggtgc gctccaagca cccacgcaag gacgtagagt
3601 ctgggttgta tctgtacgac catctgaaga accaaccgga gttgggtggc tatcagatca
3661 gcattccgca aaagggcgtg gtggataaac gcggtaaacg taaaaatcga ccagcccgca
3721 aggcgagctt gagcctgcgc agtgggcgca tcacgctaaa acaggggaat atcacgctca
3781 acgcggtgct ggccgaggag attaacccgc ccaagggtga gaccccgttg aaatggttgt
3841 tgctgaccag cgaaccggtc gagtcgctag cccaagcctt gcgcgtcatc gacatttata
3901 cccatcgctg gcggatcgag gagttccata aggcatggaa aaccggagca ggagccgaga
3961 ggcaacgcat ggaggagccg gataatctgg agcggatggt ctcgatcctc tcgtttgttg
4021 cggtcaggct gttacagctc agagaaagct tcacgctgcc gcaagcactc agggcgcaag
4081 ggctgctaaa ggaagcggaa cacgtagaaa gccagtccgc agaaacggtg ctgaccccgg
4141 atgaatgtca gctactgggc tatctggaca agggaaaacg caagcgcaaa gagaaagcag
4201 gtagcttgca gtgggcttac atggcgatag ctagactggg cggttttatg gacagcaagc
4261 gaaccggaat tgccagctgg ggcgccctct gggaaggttg ggaagccctg caaagtaaac
4321 tggatggctt tcttgccgcc aaggatctga tggcgcaggg gatcaagatc tgacccggga
4381 ctagttggtg tggaagtcca tatatcggtg atcaatatag tggttgacat gctggctagt
4441 caacattgag ccttttgatc atgcaaatat attacggtat tttacaatca aatatcaaac
4501 ttaactattg actttataac ttatttaggt ggtaacattc ttataaaaaa gaaaaaaatt
4561 actgcaaaac agtactagct tttaacttgt atcctaggtt atctatgctg tctcaccata
4621 gagaatatta cctatttcag aatgtatgtc catgattcgc cgggtaaata catataatac
4681 acaaatctgg cttaataaag tctataatat atctcataaa gaagtgctaa attggctagt
4741 gctatatatt tttaagaaaa tttcttttga ctaagtccat atcgactttg taaaagttca
4801 ctttagcata catatattac acgagccaga aattgtaact tttgcctaaa atcacaaatt
4861 gcaaaattta attgcttgca aaaggtcaca tgcttataat caactttttt aaaaatttaa
4921 aatactttttt tattttttat ttttaaacat aaatgaaata atttatttat tgtttatgat
4981 taccgaaaca taaaacctgc tcaagaaaaa gaaactgttt tgtccttgga aaaaaagcac
5041 tacctaggag cggccaaaat gccgaggctt tcatagctta aactctttac agaaaatagg
5101 cattatagat cagttcgagt tttcttattc ttccttccgg ttttatcgtc acagttttac
5161 agtaaataag tatcacctct tagagttaac tatgagataa gcaagtatca tctcatttca
5221 tttacctgaa gtcgagtaaa cagaaaatcc aattgttgat gaacctcaat gacttagaac
5281 tatctatcgg cagatcatat aaagaggatt taggtactcg agaacgctcc gtacatgcag
5341 ctaaacctcc aagcatctct aacttgggta gtgctttctc caccattttc tgtgcttgct
5401 ccttcgtggc aagtccagcc cataatgccc agaatgtagt tgcggattcg tatgacgttc
5461 tgtgcttgat ttttgtgttg tagtcaaaga aaaaccccga ctcgtcatcc cacatatatt
5521 tggtaattga tgaggcaacg ctaattatca acatatagat tgttatctat ctgcatgaac
5581 acgaaatctt tacttgacga cttgaggctg atggtgttta tgcaaagaaa ccactgtgtt
5641 taatatgtgt cactgtttga tattactgtc agcgtagaag ataatagtaa aagcggttaa
5701 taagtgtatt tgagataagt gtgataaagt ttttacagcg aaaagacgat aaatacaaga
5761 aaatgattac gaggatacgg agagaggtat gtacatgtgt atttatatac taagctgccg
5821 gcggttgttt gcaagaccga gaaaaggcta gcaagaatcg ggtcattgta gcgtatgcgc
5881 ctgtgaacat tctcttcaac aagtttgatt ccattgcggt gaaatggtaa aagtcaaccc
5941 cctgcgatgt atattttcct gtacaatcaa tcaaaaagcc aaatgattta gcattatctt
6001 tacatcttgt tattttacag attttatgtt tagatctttt atgcttgctt ttcaaaaggc
6061 ctgcaggcaa gtgcacaaac aatacttaaa taaatactac tcagtaataa cctatttctt
6121 agcattttttg acgaaatttg ctattttgtt agagtctttt acaccatttg tctccacacc
6181 tccgcttaca tcaacaccaa taacgccatt taatctaagc gcatcaccaa cattttctgg
6241 cgtcagtcca ccagctaaca taaaatgtaa gctctgcctc gcgcgtttcg gtgatgacgg
6301 tgaaaacctc tgacacatgc agctcccgga gacggtcaca gcttgtctgt aagcggatgc
6361 cgggagcaga caagcccgtc agggcgcgtc agcgggtgtt ggcgggtgtc ggggcgcagc
6421 catgacccag tcacgtagcg atagcggagt gtatactggc ttaactatgc ggcatcagag
6481 cagattgtac tgagagtgca ccatatgcgg tgtgaaatac cgcacagatg cgtaaggaga
6541 aaataccgca tcaggcgctc ttccgcttcc tcgctcactg actcgctgcg ctcggtcgtt
6601 cggctgcggc gagcggtatc agctcactca aaggcggtaa tacggttatc cacagaatca
6661 ggggataacg caggaaagaa catgtgagca aaaggccagc aaaaggccag gaaccgtaaa
6721 aaggccgcgt tgctggcgtt tttccatagg ctccgccccc ctgacgagca tcacaaaaat
```

```
 6781 cgacgctcaa gtcagaggtg gcgaaacccg acaggactat aaagatacca ggcgtttccc
 6841 cctggaagct ccctcgtgcg ctctcctgtt ccgaccctgc cgcttaccgg atacctgtcc
 6901 gcctttctcc cttcgggaag cgtggcgctt tctcatagct cacgctgtag gtatctcagt
 6961 tcggtgtagg tcgttcgctc caagctgggc tgtgtgcacg aaccccccgt tcagcccgac
 7021 cgctgcgcct tatccggtaa ctatcgtctt gagtccaacc cggtaagaca cgacttatcg
 7081 ccactggcag cagccactgg taacaggatt agcagagcga ggtatgtagg cggtgctaca
 7141 gagttcttga agtggtggcc taactacggc tacactagaa ggacagtatt tggtatctgc
 7201 gctctgctga agccagttac cttcggaaaa agagttggta gctcttgatc cggcaaacaa
 7261 accaccgctg gtagcggtgg tttttttgtt tgcaagcagc agattacgcg cagaaaaaaa
 7321 ggatctcaag aagatccttt gatcttttct acggggtctg acgctcagtg gaacgaaaac
 7381 tcacgttaag ggattttggt catgagatta tcaaaaagga tcttcaccta gatcctttta
 7441 aattaaaaat gaagttttaa atcaatctaa agtatatatg agtaaacttg gtctgacagg
 7501 agctcgctca gaagaactcg tcaagaaggc gatagaaggc gatgcgctgc gaatcgggag
 7561 cggcgatacc gtaaagcacg aggaagcggt cagcccattc gccgccaagc tcttcagcaa
 7621 tatcacgggt agccaacgct atgtcctgat agcggtccgc cacacccagc cggccacagt
 7681 cgatgaatcc agaaaagcgg ccattttcca ccatgatatt cggcaagcag gcatcgccat
 7741 gggtcacgac gagatcctcg ccgtcgggca tccgcgcctt gagcctggcg aacagttcgg
 7801 ctggcgcgag cccctgatgc tcttcgtcca gatcatcctg atcgacaaga ccggcttcca
 7861 tccgagtacg tgctcgctcg atgcgatgtt tcgcttggtg gtcgaatggg caggtagccg
 7921 gatcaagcgt atgcagccgc cgcattgcat cagccatgat ggatactttc tcggcaggag
 7981 caaggtgaga tgacaggaga tcctgccccg gcacttcgcc caatagcagc cagtcccttc
 8041 ccgcttcagt gacaacgtcg agcacagctg cgcaaggaac gcccgtcgtg gccagccacg
 8101 atagccgcgc tgcctcgtct tggagttcat tcagggcacc ggacaggtcg gtcttgacaa
 8161 aaagaaccgg gcgcccctgc gctgacagcc ggaacacggc ggcatcagag cagccgattg
 8221 tctgttgtgc ccagtcatag ccgaatagcc tctccaccca agcggccgga gaacctgcgt
 8281 gcaatccatc ttgttcaatc atgcgaaacg atcctcatcc tgtctcttga tcagatcttg
 8341 atcccctgcg ccatcagatc cttggcggca agaaagccat ccagtttact ttgcagggct
 8401 tcccaacctt accagagggc gccccagctg gcaattccgg ttcgcttgct gtccataaaa
 8461 ccgcccagtc tagctatcgc catgtaagcc cactgcaagc tacctgcttt ctctttgcgc
 8521 ttgcgttttc ccttgtccag atagcccagt agctgacatt catccgggcg atcgatggaa
 8581 gccggcggca cctcgctaac ggattcacca ctccaagaat tggagccaat caattcttgc
 8641 ggagaactgt gaatgcgcaa accaaccctt ggcagaacat atccatcgcg tccgccatct
 8701 ccagcagccg cacgcggcgc atcggggggg gggtttcaat tcaattcatc attttttttt
 8761 tattcttttt tttgatttcg gtttctttga aatttttttg attcggtaat ctccgaacag
 8821 aaggaagaac gaaggaagga gcacagactt agattggtat atatacgcat atgtagtgtt
 8881 gaagaaacat gaaattgccc agtattctta acccaactgc acagaacaaa aacctgcagg
 8941 aaacgaagat aaatcatgtc gaaagctaca tataaggaac gtgctgctac tcatcctagt
 9001 cctgttgctg ccaagctatt taatatcatg cacgaaaagc aaacaaactt gtgtgcttca
 9061 ttggatgttc gtaccaccaa ggaattactg gagttagttg aagcattagg tcccaaaatt
 9121 tgtttactaa aaacacatgt ggatatcttg actgattttt ccatggaggg cacagttaag
 9181 ccgctaaagg cattatccgc caagtacaat tttttactct tcgaagacag aaaatttgct
 9241 gacattggta atacagtcaa attgcagtac tctgcgggtg tatacagaat agcagaatgg
 9301 gcagacatta cgaatgcaca cggtgtggtg ggcccaggta ttgttagcgg tttgaagcag
 9361 gcggcagaag aagtaacaaa ggaacctaga ggccttttga tgttagcaga attgtcatgc
 9421 aagggctccc tatctactgg agaatatact aagggtactg ttgacattgc gaagagcgac
 9481 aaagattttg ttatcggctt tattgctcaa agagacatgg gtggaagaga tgaaggttac
 9541 gattggttga ttatgacacc cggtgtgggt ttagatgaca agggagacgc attgggtcaa
 9601 cagtatagaa ccgtggatga tgtggtctct acaggatctg acattattat tgttggaaga
 9661 ggactatttg caaagggaag ggatgctaag gtagagggtg aacgttacag aaaagcaggc
 9721 tgggaagcat atttgagaag atgcggccag caaaactaaa aaactgtatt ataagtaaat
 9781 gcatgtatac taaactcaca aattagagct tcaatttaat tatatcagtt attacccggg
 9841 aatctcggtc gtaatgattt ttataatgac gaaaaaaaaa aaattggaaa gaaaaccccc
 9901 cccccgcagc gttgggtcct ggccacgggt gcgcatgatc gtgctcctgt cgttgaggac
 9961 ccggctaggc tggcggggtt gccttactgg ttagcagaat gaatcaccga tacgcgagcg
10021 aacgtgaagc gactgctgct gcaaaacgtc tgcgacctga gcaacaacat gaatggtctt
10081 cggtttccgt gtttcgtaaa gtctggaaac gcggaagtca gcgccctgca ccattatgtt
10141 ccggatctgc atcgcaggat gctgctggct accctgtgga acacctacat ctgtattaac
10201 gaagcgctgg cattgaccct gagtgatttt tctctggtcc cgccgcatcc ataccgccag
10261 ttgtttaccc tcacaacgtt ccagtaaccg ggcatgttca tcatcagtaa cccgtatcgt
10321 gagcatcctc tctcgtttca tcggtatcat tacccccatg aacagaaatt cccccttaca
10381 cggaggcatc aagtgaccaa acaggaaaaa accgccctta acatggcccg ctttatcaga
10441 agccagacat taacgcttct ggagaaactc aacgagctgg acgcggatga acaggcagac
10501 atctgtgaat cgcttcacga ccacgctgat gagctttacc gcaggtgg
//
```

Supporting table S2. Employed β-galactosidase-encoding *lacZ* gene sequence. The *lacZ* gene (*Escherichia coli* str. K-12 substr. MG1655) was obtained *via* PCR from pRcExpII2-YF1-FixJ-PFixK2lacZ (A. Loeschcke, unpublished). The gene can be included in a recombinant yTREX transposon downstream of a (promoter-less) biosynthetic gene cluster (without transcription termination sites) and this way be used to report complete transcription in *P. putida* KT2440 after random chromosomal integration *via* transposon Tn5, which can lead to positioning of the biosynthetic genes downstream of a suitable host promoter (Domröse *et al*., 2017; doi: 10.1016/j.synbio.2017.11.001). The coding sequence is underlined from start to stop codon (bold). The original *E. coli* 5'-UTR including the purine-rich region of the RBS is highlighted green.

***lacZ* gene sequence**
CACAC*AGGAAA*CAGCT**ATG**ACCATGATTACGGATTCACTGGCCGTCGTTTTACAACGTCGTGACTGGGAAAACCCTGGCGT
TACCCAACTTAATCGCCTTGCAGCACATCCCCCTTTCGCCAGCTGGCGTAATAGCGAAGAGGCCCGCACCGATCGCCCTTC
CCAACAGTTGCGCAGCCTGAATGGCGAATGGCGCTTTGCCTGGTTTCCGGCACCAGAAGCGGTGCCGGAAAGCTGGCTG
GAGTGCGATCTTCCTGAGGCCGATACTGTCGTCGTCCCCTCAAACTGGCAGATGCACGGTTACGATGCGCCCATCTACAC
CAACGTGACCTATCCCATTACGGTCAATCCGCCGTTTGTTCCCACGGAGAATCCGACGGGTTGTTACTCGCTCACATTTAAT
GTTGATGAAAGCTGGCTACAGGAAGGCCAGACGCGAATTATTTTTGATGGCGTTAACTCGGCGTTTCATCTGTGGTGCAAC
GGGCGCTGGGTCGGTTACGGCCAGGACAGTCGTTTGCCGTCTGAATTTGACCTGAGCGCATTTTTACGCGCCGGAGAAAA
CCGCCTCGCGGTGATGGTGCTGCGCTGGAGTGACGGCAGTTATCTGGAAGATCAGGATATGTGGCGGATGAGCGGCATTT
TCCGTGACGTCTCGTTGCTGCATAAACCGACTACACAAATCAGCGATTTCCATGTTGCCACTCGCTTTAATGATGATTTCAG
CCGCGCTGTACTGGAGGCTGAAGTTCAGATGTGCGGCGAGTTGCGTGACTACCTACGGGTAACAGTTTCTTTATGGCAGG
GTGAAACGCAGGTCGCCAGCGGCACCGCGCCTTTCGGCGGTGAAATTATCGATGAGCGTGGTGGTTATGCCGATCGCGTC
ACACTACGTCTGAACGTCGAAAACCCGAAACTGTGGAGCGCCGAAATCCCGAATCTCTATCGTGCGGTGGTTGAACTGCAC
ACCGCCGACGGCACGCTGATTGAAGCAGAAGCCTGCGATGTCGGTTTCCGCGAGGTGCGGATTGAAAATGGTCTGCTGCT
GCTGAACGGCAAGCCGTTGCTGATTCGAGGCGTTAACCGTCACGAGCATCATCCTCTGCATGGTCAGGTCATGGATGAGC
AGACGATGGTGCAGGATATCCTGCTGATGAAGCAGAACAACTTTAACGCCGTGCGCTGTTCGCATTATCCGAACCATCCGC
TGTGGTACACGCTGTGCGACCGCTACGGCCTGTATGTGGTGGATGAAGCCAATATTGAAACCCACGGCATGGTGCCAATG
AATCGTCTGACCGATGATCCGCGCTGGCTACCGGCGATGAGCGAACGCGTAACGCGAATGGTGCAGCGCGATCGTAATCA
CCCGAGTGTGATCATCTGGTCGCTGGGGAATGAATCAGGCCACGGCGCTAATCACGACGCGCTGTATCGCTGGATCAAAT
CTGTCGATCCTTCCCGCCCGGTGCAGTATGAAGGCGGCGGAGCCGACACCACGGCCACCGATATTATTTGCCCGATGTAC
GCGCGCGTGGATGAAGACCAGCCCTTCCCGGCTGTGCCGAAATGGTCCATCAAAAAATGGCTTTCGCTACCTGGAGAGAC
GCGCCCGCTGATCCTTTGCGAATACGCCCACGCGATGGGTAACAGTCTTGGCGGTTTCGCTAAATACTGGCAGGCGTTTC
GTCAGTATCCCCGTTTACAGGGCGGCTTCGTCTGGGACTGGGTGGATCAGTCGCTGATTAAATATGATGAAAACGGCAACC
CGTGGTCGGCTTACGGCGGTGATTTTGGCGATACGCCGAACGATCGCCAGTTCTGTATGAACGGTCTGGTCTTTGCCGAC
CGCACGCCGCATCCAGCGCTGACGGAAGCAAAACACCAGCAGCAGTTTTTCCAGTTCCGTTTATCCGGGCAAACCATCGA
AGTGACCAGCGAATACCTGTTCCGTCATAGCGATAACGAGCTCCTGCACTGGATGGTGGCGCTGGATGGTAAGCCGCTGG
CAAGCGGTGAAGTGCCTCTGGATGTCGCTCCACAAGGTAAACAGTTGATTGAACTGCCTGAACTACCGCAGCCGGAGAGC
GCCGGGCAACTCTGGCTCACAGTACGCGTAGTGCAACCGAACGCGACCGCATGGTCAGAAGCCGGGCACATCAGCGCCT
GGCAGCAGTGGCGTCTGGCGGAAAACCTCAGTGTGACGCTCCCCGCCGCGTCCCACGCCATCCCGCATCTGACCACCAG
CGAAATGGATTTTTGCATCGAGCTGGGTAATAAGCGTTGGCAATTTAACCGCCAGTCAGGCTTTCTTTCACAGATGTGGATT
GGCGATAAAAAACAACTGCTGACGCCGCTGCGCGATCAGTTCACCCGTGCACCGCTGGATAACGACATTGGCGTAAGTGA
AGCGACCCGCATTGACCCTAACGCCTGGGTCGAACGCTGGAAGGCGGCGGGCCATTACCAGGCCGAAGCAGCGTTGTTG
CAGTGCACGGCAGATACACTTGCTGATGCGGTGCTGATTACGACCGCTCACGCGTGGCAGCATCAGGGGAAAACCTTATT
TATCAGCCGGAAAACCTACCGGATTGATGGTAGTGGTCAAATGGCGATTACCGTTGATGTTGAAGTGGCGAGCGATACACC
GCATCCGGCGCGGATTGGCCTGAACTGCCAGCTGGCGCAGGTAGCAGAGCGGGTAAACTGGCTCGGATTAGGGCCGCAA
GAAAACTATCCCGACCGCCTTACTGCCGCCTGTTTTGACCGCTGGGATCTGCCATTGTCAGACATGTATACCCCGTACGTC
TTCCCGAGCGAAAACGGTCTGCGCTGCGGGACGCGCGAATTGAATTATGGCCCACACCAGTGGCGCGGCGACTTCCAGTT
CAACATCAGCCGCTACAGTCAACAGCAACTGATGGAAACCAGCCATCGCCATCTGCTGCACGCGGAAGAAGGCACATGGC
TGAATATCGACGGTTTCCATATGGGGATTGGTGGCGACGACTCCTGGAGCCCGTCAGTATCGGCGGAATTACAGCTGAGC
GCCGGTCGCTACCATTACCAGTTGGTCTGGTGTCAAAAA**TAA**

Table S3. Assay solutions and calculations for determining Miller units. The standard assay for determining *β*-galactosidase activity in cells was originally described by Jeffrey H. Miller [Miller (1972) Experiments in Molecular Genetics, Cold Spring Harbor Laboratory Press, Cold Spring Harbor, NY; ISBN: 0-87969-106-9] which coined the term ‘Miller units’. Based on that methodology, we conducted a modified Miller assay to quantify *β*-galactosidase activity, employing the listed solutions and calculation.

**Z-buffer**<br>60 mM Na_2HPO_4<br>40 mM NaH_2PO_4<br>10 mM KCl<br>1 mM $MgSO_4$<br>50 mM β-mercaptoethanol (add prior to use)
**Diluted Z-buffer**<br>¼ Z-buffer<br>¾ dH2O
**ONPG substrate solution**<br>0.8 mg/mL ONPG in diluted Z-buffer (dilute Z-buffer prior to use)
**Stop solution**<br>1 M Na_2CO_3
**Calculation of Miller units**<br>$(A_{420} / OD_{600}) \times (V_{total} \times 1000 / V_{sample} \times t)$ = *β*-galactosidase activity [Miller units]<br>A_{420} = measured *o*-nitrophenol absorption at 420 nm<br>OD_{600} = cell density at 600 nm<br>V_{total} = total volume (1.25 mL)<br>V_{sample} = sample volume (here: 0.001 mL; higher sample volumes within the 400 µL ‘sample solution’ [cell culture sample + diluted Z-buffer] or diluted samples can be used if necessary)<br>T = time (2 min; can be prolonged up to 30 min for clones showing weak reporter gene expression)

Appendix S1. Yeast transformation protocol for cloning of yTREX vector constructs based on previously developed methodology (Gietz and Schiestl, 2007; Gietzt et al., 1995).

1. **Prepare DNA fragments that are to be assembled:** Combine the linearised vector DNA with all DNA fragments of the target gene cluster to be integrated (see section 3.2 of this manuscript) in a reaction tube to a total volume of 34 µL. We normally employ 14 µL H_2O, 5 µL yTREX vector solution and 15 µL insert solution and use equal volumes of insert solution, if multiple fragments are combined, e.g. in case of 2 insert fragments, 7.5 µL of each. The amounts of all DNA parts should be adjusted to 0.1-1 µg.

2. **Prepare yeast cells for transformation and assembly of DNA fragments:** Grow *S. cerevisiae* VL6-48 (ATCC® MYA-3666™ (Kouprina et al., 1998) on agar plates made of YPD (yeast peptone dextrose) liquid medium (10 g/L yeast extract, 20 g/L peptone, 20 g/L glucose) (Sherman, 2002) with 20 g/L Agar-Agar at 30 °C. Use fresh clones to inoculate a pre-culture in liquid YPD medium, and cultivate over night at 30 °C, shaking at 130 rpm (e.g. using 10 mL YPD medium in a 100 mL Erlenmeyer flask). Inoculate a main culture in 50 mL YPD liquid medium in a 500 mL baffled flask to an $OD_{600\,nm}$ of 0.25 and cultivate at 30 °C and shaking at 130 rpm to an $OD_{600\,nm}$ = 0.9-1, which normally takes about 5 h (adaptation to Gietz and Schiestl steps 1-4). Harvest the cells by centrifugation (4 °C, 5 min, 2900 x g) and discard the supernatant. Then, resuspend the cells in 25 mL sterile deionized water, centrifuge again (4 °C, 5 min, 2,900 x g) and discard the supernatant. Resuspend washed cells in 1 mL 100 mM LiAc (pH 8.4 – 8.9) and transfer to a 1.5 mL reaction tube. Centrifuge cells shortly (22 °C, 30 s, 16,000 x g), discard supernatant, resuspend the cells in 500 µL 100 mM LiAc and aliquot in 50 µL samples (while the use of fresh cells for maximal transformation efficiency is recommended, we have also successfully employed cell samples, that were stored at this point until use at 4 °C for up to one week, as well as cell samples that were supplemented glycerol to a final concentration of 50% and stored at -80 °C for up to four weeks before use).

3. **Transform DNA into yeast cells:** Denature an aliquot of carrier DNA (Salmon sperm single stranded DNA, 2 mg/ml in TE-buffer, e.g. by Sigma-Aldrich (Merck)) at 99 °C for 10 min and immediately chill on ice. Centrifuge an aliquot of competent yeast cells obtained in the previous step 2 (22 °C, 30 s, 16,000 x g) and discard the supernatant. Resuspend the cells in 36 µL LiAc (1M), add 240 µL PEG 3350 (50% w/v) and 50 µL carrier DNA. Then add 34 µL DNA suspension (containing the prepared DNA fragments, obtained in above described step 1). Thoroughly vortex the suspension, then subject the mixture to a heat shock by incubation at 42 °C for 30 min. Centrifuge to pellet cells (22 °C, 30 s, 16,000 x g), discard supernatant and resuspend cells in 1 mL YPD medium and incubate at 30 °C for 0.5 h – 3 h whilst shaking at 600 rpm.

4. **Plate transformation mixture:** Harvest cells from transformation mixtures (see previous step 3) by short centrifugation (22 °C, 30 s, 16,000 x g) and discard the supernatant. Gently resuspend the cells in 200 µL sterile deionized H_2O, using a 1 mL pipette (to avoid shearing forces of smaller pipette tips). Then, plate the cell suspension in different dilutions on SD^{-Ura} agar plates for selection of clones carrying the plasmid with the *URA* marker. Synthetic dextrose minimal medium (Sherman, 2002) without uracil is composed of 6.7 g/L Yeast-Nitrogen-Base (without Amino Acids) (Carl Roth®, Karlsruhe, Germany), 1.926 g/L Kaiser Uracil drop-out mixture (Kaiser et al., 1994) (Formedia™, Norfolk, United Kingdom), 20 g/L glucose, and completed with 20 g/L agar for SD^{-Ura} agar plates. We normally avoid obtaining plates with either a yeast lawn or no clones at all by using 20 µL for one plate, and 180 µL for another plate. Incubate agar plates for 2-4 days at 30 °C for clone

formation. Depending on the experiment, the resulting number of clones to expect may vary considerably. A control with vector DNA but water instead of insert DNA should be used to indicate successful assembly of insert elements into the yTREX vector rather than clone formation due to transformation of insufficiently hydrolysed vector DNA. The control plate should accordingly exhibit a significantly lower number of clones than the plates where actual assembly transformation samples were plated.

5. **Isolate plasmid DNA from yeast:** Transfer yeast colonies from plates (grown in previous step 4) to 1 mL SD^{-Ura} liquid medium and incubate in RoundwellPlates® (m2p-labs GmbH, Baesweiler, Germany) at 30 °C under constant shaking (1000 rpm) over night. Harvest cells by centrifugation and solve in resuspension buffer from innuPREP Plasmid Mini Kit (Analytik Jena AG, Jena, Germany), supplemented with 12.5 U of Zymolyase® (Zymo Research Europe GmbH, Freiburg, Germany). Incubate the solution for 2-3 h at 37 °C (the solution should become clearer during this time) and then proceed with plasmid isolation according to the manufacturer's protocol. Elution of the plasmids should be performed with maximal volume of 10 µL nuclease-free H_2O due to the low plasmid density in yeast cells.

6. **Amplify plasmid DNA in *E. coli* and verify correct assembly:** Transform the isolated plasmid DNA (obtained in previous step 5) into *E. coli* DH5α for amplification, isolation and analysis for correct assembly by commonly established methods. To this end, use LB or TB medium supplemented with 50 µg/mL kanamycin for plasmid selection during cultivation. Do not use tetracycline as *E. coli* clones which only carry the transposon would also exhibit this resistance.
